# *trans*-Fatty acids facilitate DNA damage-induced apoptosis through the mitochondrial JNK-Sab-ROS positive feedback loop

**DOI:** 10.1038/s41598-020-59636-6

**Published:** 2020-02-17

**Authors:** Yusuke Hirata, Aya Inoue, Saki Suzuki, Miki Takahashi, Ryosuke Matsui, Nozomu Kono, Takuya Noguchi, Atsushi Matsuzawa

**Affiliations:** 1 Laboratory of Health Chemistry, Graduate School of Pharmaceutical Sciences, Tohoku University, Sendai, Japan; 2 Department of Health Chemistry, Graduate School of Pharmaceutical Sciences, The University of Tokyo, Tokyo, Japan

**Keywords:** Kinases, Stress signalling, DNA damage response, Risk factors

## Abstract

*trans*-Fatty acids (TFAs) are unsaturated fatty acids that contain one or more carbon-carbon double bonds in *trans* configuration. Epidemiological evidence has linked TFA consumption with various disorders, including cardiovascular diseases. However, the underlying pathological mechanisms are largely unknown. Here, we show a novel toxic mechanism of TFAs triggered by DNA damage. We found that elaidic acid (EA) and linoelaidic acid, major TFAs produced during industrial food manufacturing (so-called as industrial TFAs), but not their corresponding *cis* isomers, facilitated apoptosis induced by doxorubicin. Consistently, EA enhanced UV-induced embryonic lethality in *C. elegans* worms. The pro-apoptotic action of EA was blocked by knocking down Sab, a c-Jun N-terminal kinase (JNK)-interacting protein localizing at mitochondrial outer membrane, which mediates mutual amplification of mitochondrial reactive oxygen species (ROS) generation and JNK activation. EA enhanced doxorubicin-induced mitochondrial ROS generation and JNK activation, both of which were suppressed by *Sab* knockdown and pharmacological inhibition of either mitochondrial ROS generation, JNK, or Src-homology 2 domain-containing protein tyrosine phosphatase 1 (SHP1) as a Sab-associated protein. These results demonstrate that in response to DNA damage, TFAs drive the mitochondrial JNK-Sab-ROS positive feedback loop and ultimately apoptosis, which may provide insight into the common pathogenetic mechanisms of diverse TFA-related disorders.

## Introduction

*trans*-Fatty acids (TFAs) are defined as unsaturated fatty acids containing one or more carbon-carbon double bonds in *trans* configuration. TFAs, such as elaidic acid (EA, C18:1 *t*9) and linoelaidic acid (LEA, C18:2 *t*9,*t*12), so-called as industrial TFAs, are produced during the food manufacturing processes, mainly through partial hydrogenation of vegetable and fish oils that contain *cis* isomers of TFAs, hereafter referred to as *cis*-fatty acids (CFAs)^1^. On the other hand, TFAs such as *trans*-vaccenic acid (TVA, C18:1 *t*11), so-called as ruminant TFAs, are produced via enzymatic isomerization of CFAs by ruminal microbes in cows and sheep, and are present in dairy products and meat^1^. Compelling epidemiological evidence has shown that the intake of TFAs, particularly industrial TFAs, increases the risk of various disorders, such as systemic inflammation, metabolic syndrome, neurodegenerative disorders, and cardiovascular diseases (CVDs)^2–5^. However, few studies have focused on the mechanisms of action of TFAs, and the molecular mechanisms underlying TFA-related disorders remain to be elucidated.

Among TFA-related disorders, TFAs have been most highly linked with atherosclerosis, one of the major cause of CVDs, and the underlying mechanisms have been explained by their deleterious effects on vascular endothelial functions and lipoprotein regulation^3,6–10^. Importantly, we have recently revealed a novel toxic function of TFAs as an enhancer of inflammatory signaling and cell death induced by extracellular ATP, one of the damage-associated molecular patterns that are leaked from injured cells and serve as potent pro-inflammatory and atherogenic factors^11^. Major food-associated TFAs, including EA, but not their corresponding *cis* isomers, promote extracellular ATP-induced apoptosis in a macrophage cell line RAW264.7, through enhancing activation of the apoptosis signal-regulating kinase 1 (ASK1)-p38 mitogen activated protein (MAP) kinase pathway downstream of the P2X purinoceptor 7 (P2X_7_)^11^. These findings provided a novel mechanistic insight into TFA-related atherosclerosis, where macrophage apoptosis in atherosclerotic lesions is the major cause of disease progression^12,13^. However, to date, extracellular ATP-induced apoptosis via the P2X_7_-ASK1-p38 axis has been observed in limited cell types, such as macrophages^11,14^, and it is therefore speculated that another pathogenetic mechanism is shared with not only atherosclerosis, but also other diverse TFA-related disorders.

DNA is vulnerable to many kinds of endogenous and environmental stresses, such as DNA replication, reactive oxygen species (ROS), and genotoxins (e.g. UV, ionizing radiation, and anti-cancer drugs), which induce a variety of DNA lesions^15^. Since DNA lesions cause genomic instability and gene mutations that lead to various diseases including cancer, cells sense and counteract DNA damage by a mechanism referred to as DNA damage response (DDR); when DNA damage level is low, cells repair DNA lesions and maintain survival, whereas when DNA damage is beyond repair, cells elicit senescence or programmed cell death including apoptosis^16^. Dysregulation of DDR causes aberrant gene regulation and cellular malfunctions, which links to diverse human diseases, such as inflammatory diseases, metabolic syndromes, neurodegenerative disorders, and CVDs, that are also associated with the intake of TFAs^17^. Taken together, it is assumed that TFAs may disrupt DDR signaling, and thereby contribute to the pathogenesis and development of TFA-related disorders, although no study has addressed this assumption.

In this study, we showed that EA and LEA, the most abundant industrial TFAs in foods^1^, but not their corresponding *cis* isomers or palmitic acid (PA, C16:0) as a typical saturated fatty acid, potentiated cell death induced by DNA-damaging agent doxorubicin (Dox) in multiple types of cell lines including RAW264.7 cells, U2OS cells, and HeLa cells. EA enhanced DNA damage-induced mitochondrial ROS generation and activation of the stress-responsive MAP kinases, c-Jun N-terminal kinase (JNK) and p38, through feedforward activation of the mitochondrial JNK-Sab pathway, thereby promoting apoptosis. These results demonstrate a TFA-specific pro-apoptotic effect on diverse cell types during DNA damage, which explains the common pathogenesis and progression of various TFA-related disorders.

## Results

### TFAs specifically promote DNA damage-induced apoptosis

**Figure 1:**
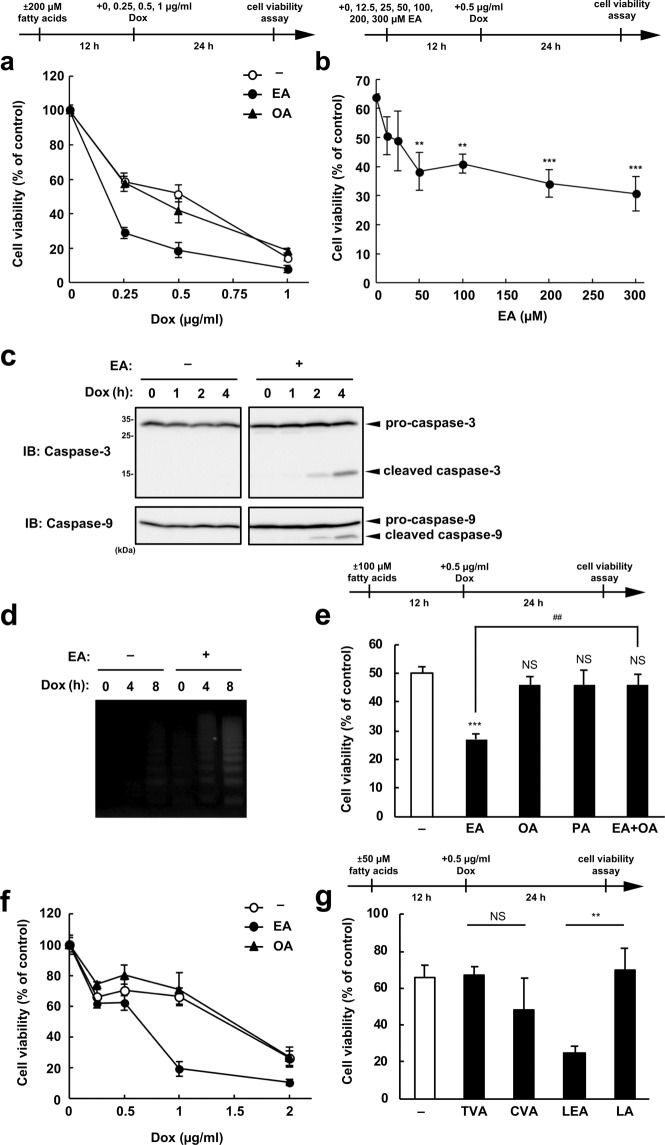
TFAs specifically promote DNA damage-induced apoptosis. (**a**) RAW264.7 cells were pretreated with or without 200 μM OA or EA for 12 h, and then stimulated with various concentrations of Dox for 24 h, subjected to cell viability assay. Data shown are the mean ± SD. (**b**) RAW264.7 cells were treated with the indicated concentrations of EA for 12 h, and then stimulated with 0.5 µg/ml Dox for 24 h, subjected to cell viability assay. Data shown are the mean ± SD. Significant differences were determined by one-way ANOVA, followed by Tukey-Kramer test: **p < 0.01; ***p < 0.001. (**c**) RAW264.7 cells were pretreated with or without 200 µM EA for 12 h, and then stimulated with 0.5 µg/ml Dox for the indicated time periods. Cell lysates were subjected to immunoblotting with the indicated antibodies. Images are cropped for clarity; full-length blots are presented in Supplementary Fig. 4a. (**d**) RAW264.7 cells were pretreated with or without 200 µM EA for 12 h, and then treated with 0.5 μg/ml Dox for 4 or 8 h, subjected to DNA fragmentation assay. (**e**) RAW264.7 cells were pretreated with 100 μM PA, OA, or EA for 12 h, and then stimulated with 0.5 µg/ml Dox for 24 h, subjected to cell viability assay. Data shown are the mean ± SD. Significant differences were determined by one-way ANOVA, followed by Tukey-Kramer test: ***p < 0.001; NS, not significant (versus control cells without fatty acid); ^##^p < 0.01. (**f**) U2OS cells were pretreated with or without 200 μM OA or EA for 12 h, and then stimulated with various concentrations of Dox for 24 h, subjected to cell viability assay. (**g**) RAW264.7 cells were pretreated with 50 μM TVA, *cis*-vaccenic acid (CVA, C18:1 *c*11), LEA or LA for 12 h, and then stimulated with 0.5 µg/ml Dox for 24 h, subjected to cell viability assay. Data shown are the mean ± SD. Significant differences were determined by two-tailed unpaired Student’s t-test: **p < 0.01; NS, not significant.

We first examined whether EA, the most abundant TFA in foods, affects DNA damage-induced cell death in RAW264.7 cells, using a DNA-damaging agent Dox. As shown in Fig. 1a, Dox treatment induced decrease in cell viability, which was potently enhanced by pretreatment of EA, but not by that of its *cis* isomer oleic acid (OA, C18:1 *c*9). When Dox concentration was fixed at 0.5 µg/ml, EA decreased cell viability in a dose-dependent manner up to 300 µM (Fig. 1b), at which no cytotoxic effect was observed^11^. In EA-pretreated cells, hallmarks of apoptosis, such as caspase-3 cleavage (activation) (Fig. 1c) and DNA ladder formation (Fig. 1d), were substantially increased in response to Dox, indicating that EA promotes Dox-induced apoptosis. We previously showed that EA promotes extracellular ATP-induced cell death, which was not suppressed by co-treatment of OA^11^. In addition, PA, a typical saturated fatty acid, also promoted ATP-induced cell death, but to a lesser extent than EA^11^. On the other hand, in the case of Dox-induced cell death, OA almost completely suppressed the pro-apoptotic effect of EA, and PA hardly promoted cell death (Fig. 1e). These data suggest that EA specifically promotes apoptosis induced by DNA damage, possibly via another different mechanism from that observed in extracellular ATP-induced cell death. The EA-specific pro-apoptotic effect was also observed in other types of cells, such as U2OS cells (Fig. 1f), HeLa cells, and HUVECs (Human umbilical vein endothelial cells) (see Supplementary Fig. S1a,b), and also when other DNA-damaging agents, such as etoposide^18^ and cisplatin^19^, were used instead of Dox (see Supplementary Fig. S1c,d). These data indicate that EA promotes cell death induced by multiple DNA-damaging agents in several cell types, supporting the generality of the pro-apoptotic effect specific for EA. Moreover, we found that Dox-induced cell death was also elevated by another major industrial TFA in foods, LEA, but not by its *cis* isomer, linoleic acid (LA, C18:2 *c*9,*c*12) or the most abundant ruminant TFA in foods, TVA (Fig. 1g), implying an importance of n-9 *trans* double bond common between EA and LEA in their pro-apoptotic action. Collectively, these results indicate that industrial TFAs, such as EA and LEA, promote DNA damage-induced apoptosis.

### TFAs promote Dox-induced apoptosis independently of p53 and TNFR1

**Figure 2:**
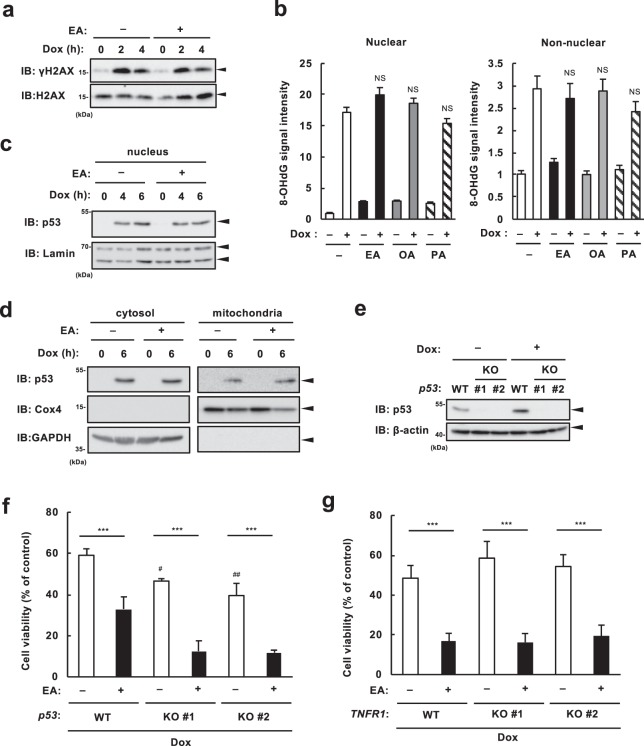
Canonical DNA damage-induced cell death pathways do not involve the cell death enhancement by EA. (**a**) U2OS cells were pretreated with or without 200 µM EA for 12 h, and then stimulated with 1 µg/ml Dox for the indicated time periods. Cell lysates were subjected to immunoblotting with the indicated antibodies. (**b**) RAW264.7 cells were pretreated with 100 µM PA, OA, or EA for 12 h, and then stimulated with 1 µg/ml Dox for 0 or 2 h. Cells were subjected with immunocytochemistry using antibodies against 8-OHdG and β-actin, and stained with DAPI for the visualization of nuclei. 8-OHdG signal intensity at nuclear and non-nuclear areas was quantified and represented as mean ± SEM (n = 20). Significant differences were determined by one-way ANOVA, followed by Tukey-Kramer test: NS, not significant (versus Dox-treated cells without any fatty acid). (c and d) U2OS cells were pretreated with or without 200 µM EA for 12 h, and then stimulated with 1 µg/ml (**c**) or 0.5 µg/ml (**d**) Dox for the indicated time periods. The nuclear extract (**c**) and the cytosol and mitochondrial fraction (**d**) were subjected to immunoblotting with the indicated antibodies. (**e**) *p53* WT and KO RAW264.7 cells were treated with or without 0.5 µg/ml Dox for 3 h. Cell lysates were subjected to immunoblotting with the indicated antibodies. (**f**) *p53* WT and KO RAW 264.7 cells were pretreated with or without 200 μM EA for 12 h, and then treated with 0.5 µg/ml Dox for 24 h, subjected to cell viability assay. Data shown are the mean ± SD. Significant differences were determined by one-way ANOVA, followed by Tukey-Kramer test: ***p < 0.001; ^#^p < 0.05; ^##^p < 0.01 (vs WT cells without EA). (**g**) *TNFR1* WT and KO U2OS cells were pretreated with or without 200 µM EA for 12 h and then treated with 1 µg/ml Dox for 24 h, subjected to cell viability assay. Data shown are the mean ± SD. Significant differences were determined by one-way ANOVA, followed by Tukey-Kramer test: ***p < 0.001.

To elucidate the underlying mechanisms of the EA-mediated pro-apoptotic effect during DNA damage, we first checked whether EA increases DNA damage itself, namely, the extent of DNA damage. As shown in Fig. 2a and Supplementary Fig. S2a, Dox-induced phosphorylation of histone 2AX (known as γ-H2AX), a widely used DNA damage marker, was comparable irrespective of the presence or absence of EA. Moreover, pretreatment of fatty acids, including EA, OA, and PA, did not affect Dox-induced accumulation of 8-hydroxy-2′-deoxyguanosine (8-OHdG), a common indicator of DNA damage related to oxidative stress, at the nuclear and the non-nuclear areas (i.e. mitochondria) (Fig. 2b and Supplementary Fig. S2b). These data suggest that EA promotes apoptosis by targeting a molecule that mediates cell death downstream of DNA damage. DNA damage induces p53-dependent mitochondrial apoptosis though the caspase-9/caspase-3 axis, which is the major cell death pathway activated during DNA damage^16^. Since we observed clear increase in Dox-induced activation of both caspase-9 and caspase-3 in EA-pretreated cells (Fig. 1c), we investigated the role of p53 in the pro-apoptotic action of EA. Immunoblot analysis showed no significant difference in Dox-induced expression of p53 at the nucleus, cytosol, and mitochondria between cells with or without EA (Fig. 2c,d). We established p53 knockout (KO) RAW264.7 cells using the CRISPR/CRISPR-associated protein 9 (Cas9) system (Fig. 2e). We unexpectedly found that p53 deficiency significantly promoted Dox-induced cell death, but we still observed that EA promoted cell death in p53 KO cells to the similar level as that in wild type (WT) cells (Fig. 2f), suggesting that p53-dependent mitochondrial apoptosis is not involved in the enhancement of cell death by EA. Another common DNA damage-induced cell death pathway requires the autocrine tumor necrosis factor-α (TNF-α)-TNF receptor 1 (TNFR1) feedforward signaling, which is triggered by DNA damage-induced NF-κB activation and subsequent TNF-α upregulation and secretion, independently of p53^20^. In order to examine the association of this pathway with the EA-specific pro-apoptotic effect, we established TNFR1 KO U2OS cells. We observed that EA promotes Dox-induced cell death in TNFR1 KO cells, comparably to WT cells (Fig. 2g). These results indicate that canonical DNA damage-induced cell death pathways, such as p53 and TNF-α-TNFR1 signaling pathways, do not involve the pro-apoptotic effect of EA.

### EA promotes DNA damage-induced cell death through mutual enhancement of JNK activation and mitochondrial ROS generation

**Figure 3:**
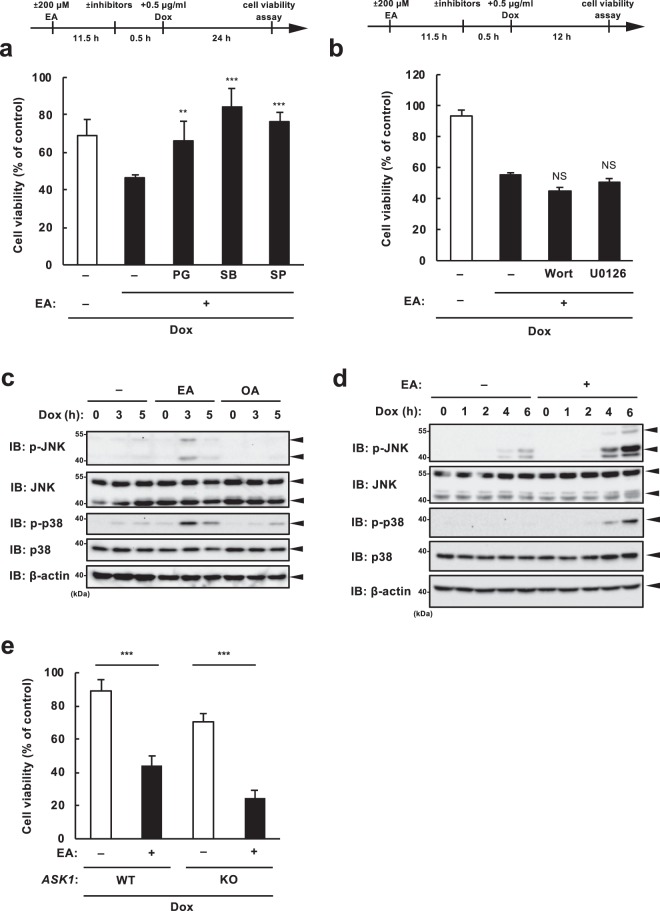
EA promotes DNA damage-induced cell death through hyperactivation of MAP kinases in a ROS-dependent and ASK1-independent manner. (**a**) RAW264.7 cells were pretreated with 200 μM EA for 12 h, and then various inhibitors including the antioxidant propyl gallate (PG, 20 μM), p38 inhibitor SB203580 (SB, 5 μM), and JNK inhibitor SP600125 (SP, 5 μM) were treated for 30 min before 0.5 μg/ml Dox treatment for 24 h, subjected to cell viability assay. Data shown are the mean ± SD. Significant differences were determined by one-way ANOVA, followed by Tukey-Kramer test: **p < 0.01; ***p < 0.001 (versus EA-pretreated cells without any inhibitor). (**b**) U2OS cells were pretreated with or without 100 μM EA for 12 h, and then treated with either 1 µM wortmannin (Wort, PI3K inhibitor) or 1µM U0126 (MEK inhibitor) for 0.5 h before 0.5 μg/ml Dox treatment for 12 h, subjected to cell viability assay. Data shown are the mean ± SD. Significant differences were determined by one-way ANOVA, followed by Tukey-Kramer test: NS, not significant (versus EA-pretreated cells without any inhibitor). (c and d) RAW264.7 cells (**c**) and U2OS cells (**d**) were pretreated with or without 200 μM OA or EA for 12 h, and then stimulated with Dox (RAW264.7 cells, 0.5 µg/ml; U2OS cells, 1 µg/ml) for the indicated time periods. Cell lysates were subjected to immunoblotting with the indicated antibodies. (**e**) *ASK1* WT and KO RAW264.7 cells were pretreated with or without 200 μM EA for 12 h, and then treated with 0.5 µg/ml Dox for 24 h, subjected to cell viability assay. Significant differences were determined by one-way ANOVA, followed by Tukey-Kramer test: ***p < 0.001.

EA promotes extracellular ATP-induced cell death through enhancing ROS-dependent activation of the ASK1-p38 pathway^11^. Dox has been reported to induce ROS generation and subsequent ASK1-p38/JNK activation, leading to cell death^21^. Taken together, we speculated that ASK1 may play a role in the EA-mediated pro-apoptotic effect observed during DNA damage. We first examined whether ROS and MAP kinases contribute to the EA-mediated cell death enhancement. Treatment of either ROS scavenger propyl gallate (PG), p38 inhibitor SB203850 (SB), or JNK inhibitor SP600125 (SP) significantly suppressed Dox-induced cell death in the presence of EA (Fig. 3a). Protein kinase B (known as Akt) and extracellular signal-regulated kinase (ERK) are well-known protein kinases that play significant roles in DDR^22^. However, neither the phosphoinositide 3-kinase (PI3K) inhibitor wortmannin (wort) nor the MAPK/ERK kinase (MEK) inhibitor U0126, widely used as inhibitors for the upstream kinases of Akt and ERK, respectively, hardly suppressed pro-apoptotic effect of EA (Fig. 3b). These results suggest that ROS, p38, and JNK particularly involve cell death enhancement by EA. Indeed, immunoblot analysis showed that EA pretreatment enhanced p38 and JNK activation in response to Dox (Fig. 3c,d). Nevertheless, cell viability assay revealed that EA-dependent decrease in cell survival was not reversed by ASK1 deficiency (Fig. 3e). These data collectively suggest that hyperactivation of the p38 and JNK MAP kinases contribute to the EA-mediated pro-apoptotic effect in a manner dependent on ROS, but independent on ASK1.

**Figure 4:**
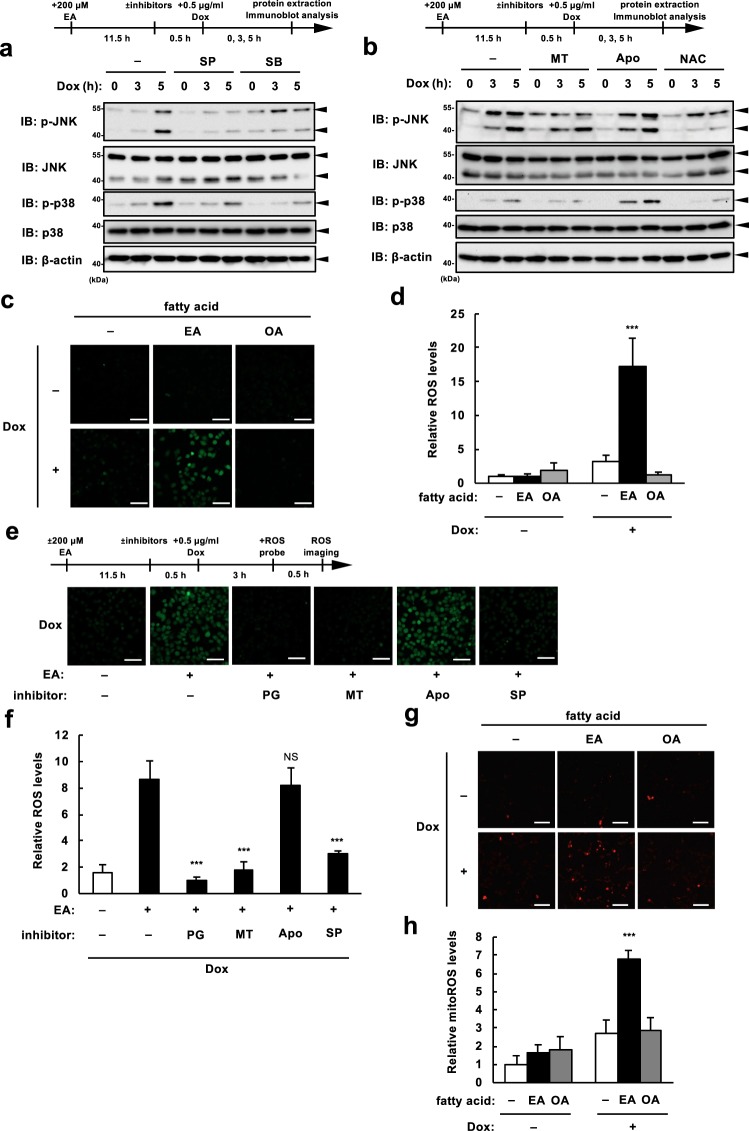
EA induces mutual enhancement of JNK activation and mitochondrial ROS generation in response to DNA damage. (**a**) RAW264.7 cells were pretreated with 200 μM EA for 12 h, and treated with either 5 μM SB or 5 µM SP 30 min before stimulation with 0.5 µg/ml Dox for the indicated time periods. Cell lysates were subjected to immunoblotting with the indicated antibodies. (**b**) RAW264.7 cells were pretreated with 200 μM EA for 12 h, and treated with either 100 μM Apocynin (Apo), 10 μM Mito-tempo (MT) and 1 mM N-acetylcysteine (NAC) 30 min before stimulation with 0.5 µg/ml Dox for the indicated time periods. Cell lysates were subjected to immunoblotting with the indicated antibodies. (**c**,**d**) RAW264.7 cells were pretreated with 200 µM EA for 12 h, and then stimulated with 0.5 μg/ml Dox for 3 h, followed by incorporation of a ROS-sensitive fluorescent probe DCFH-DA for 30 min. Green fluorescence was observed by confocal microscope (**c**), and relative ROS levels were calculated as described in Materials and Methods, shown as mean ± SD (normalized to the ROS level in the cells without fatty acid and Dox) (**d**). Scale bar, 50 µm. Significant differences were determined by one-way ANOVA, followed by Tukey-Kramer test: ***p < 0.001 (versus Dox-treated cells without fatty acid pretreatment). (**e**,**f**) RAW264.7 cells were pretreated with or without 200 μM EA for 12 h, treated with either 20 μM propyl gallate (PG), 10 μM MT, 100 μM Apo, or 5 μM SP for 30 min, and then stimulated with 0.5 μg/ml Dox for 3 h, followed by incorporation of a ROS-sensitive fluorescent probe DCFH-DA for 30 min. Green fluorescence was observed by confocal microscope (**e**), and relative ROS levels were calculated as described in Materials and Methods, shown as mean ± SD (normalized to the ROS level in the cells without EA and inhibitor) (**f**). Scale bar, 50 µm. Significant differences were determined by one-way ANOVA, followed by Tukey-Kramer test: ***p < 0.001; NS, not significant (versus EA-pretreated cells without any inhibitor). (**g,h**) U2OS cells were pretreated 200 µM EA for 12 h, and then stimulated with 0.5 μg/ml Dox for 4 h, followed by incorporation of MitoSOX Red for 30 min. Red fluorescence was observed and calculated as in (**c**,**d**). Scale bar, 100 µm. Significant differences were determined by one-way ANOVA, followed by Tukey-Kramer test: ***p < 0.001 (versus Dox-treated cells without fatty acid pretreatment).

To further clarify the molecular mechanisms underlying pro-apoptotic function of EA, we investigated the relationship between ROS, p38, and JNK. Immunoblot analysis showed that, in EA-pretreated cells, JNK inhibition diminished DNA damage-induced activation of both JNK and p38, while p38 inhibition diminished the activation of p38, but not JNK (Fig. 4a), suggesting that JNK functions upstream of p38 in the EA-mediated pro-apoptotic signaling pathway. Major sources of intracellular ROS are NADPH oxidases and mitochondria^23^. We therefore investigated whether and which source of ROS contributes to the MAP kinase activation enhanced by EA. We found that treatment of either a ROS scavenger, N-acetylcysteine (NAC) or a mitochondria-specific antioxidant, mitoTEMPO (MT) apparently decreased p38 and JNK activation, whereas treatment of an NADPH oxidase inhibitor, apocynin (Apo) did not (Fig. 4b), implying a crucial role of mitochondrial ROS (mitoROS) in EA-mediated hyperactivation of MAP kinases. We then examined whether EA affects Dox-induced ROS generation using a fluorescent ROS indicator, 2′,7′-Dichlorodihydrofluorescin diacetate (DCFH-DA). EA-pretreated cells, but not untreated and OA-pretreated cells, showed drastic increase in green fluorescence in response to Dox (Fig. 4c,d), indicating that EA specifically augments DNA damage-induced ROS generation. Further ROS measurement revealed that EA-dependent ROS generation increase was almost completely suppressed in the presence of PG or MT, but not Apo, suggesting that EA promotes mitoROS generation induced by DNA damage (Fig. 4e,f). Indeed, mitoROS measurement using the mitoROS indicator MitoSOX Red confirmed that EA pretreatment drastically increases Dox-induced mitoROS generation (Fig. 4g,h). Interestingly, we found that JNK inhibitor also potently suppressed the ROS overgeneration (Fig. 4e,f), while ROS scavenger inhibited EA-mediated JNK hyperactivation as well (Fig. 4b). Overall, these data suggest that EA mediates mutual enhancement of JNK activation and mitoROS generation induced by DNA damage, resulting in p38 hyperactivation.

### EA promotes ROS generation and pro-apoptotic signaling through the mitochondrial JNK-Sab axis

**Figure 5:**
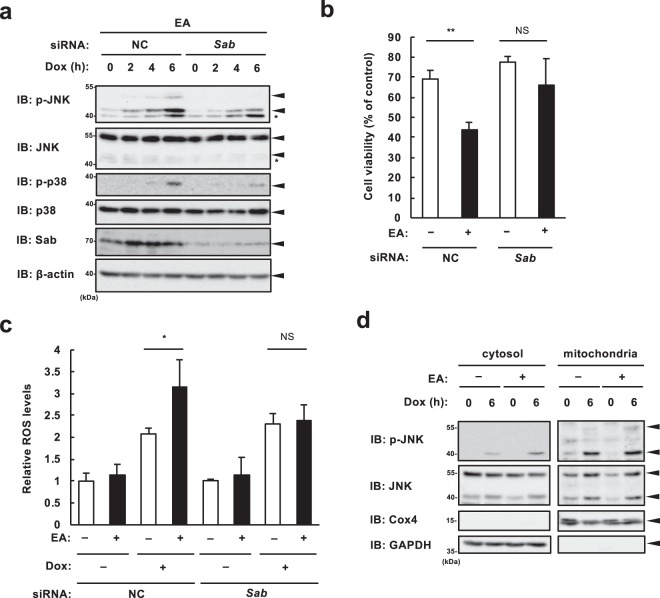
Mitochondrial JNK adaptor protein Sab is required for the pro-apoptotic action of EA. (**a**) U2OS cells were transfected with non-targeting control siRNA (NC) or siRNA targeting *Sab* for 24 h, pretreated with 200 μM EA for 12 h, and then stimulated with 1 μg/ml Dox for the indicated time periods. Cell lysates were subjected to immunoblotting with the indicated antibodies.*, non-specific band. (**b**) U2OS cells were transfected with NC or *Sab* siRNA for 36 h, reseeded and pretreated with or without 200 μM EA for 12 h. After pretreatment, cells were treated with 1 μg/ml Dox for 24 h, and subjected to cell viability assay. Data shown are the mean ± SD. Significant differences were determined by one-way ANOVA, followed by Tukey-Kramer test: *p < 0.05; NS, not significant. (**c**) U2OS cells were pretreated 200 µM EA for 12 h, and then stimulated with 1 μg/ml Dox for 8 h, followed by incorporation of a ROS-sensitive fluorescent probe DCFH-DA for 30 min. Relative ROS levels are calculated and shown as in Fig. 4d (normalized to the ROS level in NC-treated cells without EA and Dox). Significant differences were determined by two-tailed unpaired Student’s t-test: *p < 0.05; NS, not significant. (**d**) U2OS cells were pretreated 200 µM EA for 12 h, and then stimulated with 0.5 μg/ml Dox for 6 h. Cytosol and mitochondrial fractions were obtained from the cell lysates, and subjected to immunoblotting with the indicated antibodies. Images are cropped for clarity; full-length blots are presented in Supplementary Fig. 4k.

Feedforward amplification of the JNK/mitoROS loop is mediated by the mitochondrial JNK adaptor protein Sab (also referred to as SH3 domain-binding protein 5, SH3BP5)^24^. In response to various types of stresses including DNA damage, a subset of activated JNK translocates to the mitochondria, where it binds to Sab on the outer mitochondrial membrane, and thereby enforces mitoROS generation through inhibition of electron transport; overgenerated mitoROS, in turn, enhances and sustains JNK activation, leading to increased cell death^25–27^. In order to determine the role of Sab in the EA-mediated pro-apoptotic signaling, we first tried to establish and utilize Sab KO cells. However, we could not obtain cell lines completely lacking Sab in U2OS cells. We barely obtained several lines of Sab KO RAW264.7 cells, however, these cell lines constantly undergo cell death in a normal culture condition, even though they can proliferate slowly, implying a significant role of Sab in basic cellular functions. Therefore, in the following experiments, we utilized *Sab* knockdown U2OS cells (Fig. 5a), in which no apparent cytotoxicity was observed (data not shown). Immunoblot analysis showed that *Sab* knockdown inhibits not only DNA damage-induced hyperactivation of JNK, but also that of p38 mediated by EA (Fig. 5a), in line with the notion that p38 functions downstream of the JNK/mitoROS feedforward loop (Fig. 4). Moreover, we found that, in *Sab* knockdown cells, EA-dependent enhancement of both DNA damage-induced cell death (Fig. 5b) and ROS generation (Fig. 5c) was suppressed. These data collectively suggest an essential role of Sab in the EA-mediated pro-apoptotic signaling and mitoROS generation. To clarify the underlying mechanism by which EA facilitates activation of the mitochondrial JNK-Sab axis, we investigated whether EA promotes Sab-mediated mitochondrial translocation of JNK in response to DNA damage. Immunoblot analysis revealed that Dox treatment induced accumulation of JNK (also, activated JNK) in the mitochondrial fraction to the same level regardless of the presence or absence of EA (Fig. 5d), suggesting that a target of EA exists downstream of Sab after DNA damage-induced JNK translocation, and thereby facilitates mitoROS generation.

### SHP1 participates in the DNA damage-induced pro-apoptotic signaling mediated by EA

**Figure 6:**
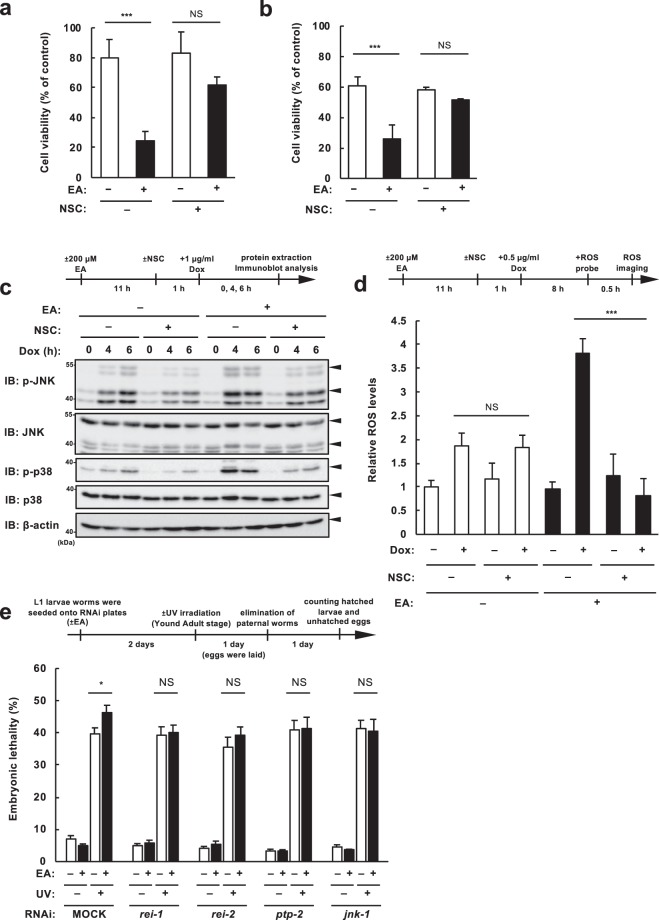
SHP1 participates in the EA-mediated pro-apoptotic signaling. (**a**,**b**) RAW264.7 cells (**a**) and U2OS cells (**b**) were pretreated with or without 200 μM EA for 12 h, treated with 10 µM NSC87877 (NSC) for 1 h, and then stimulated with 0.5 μg/ml (**a**) or 1 µg/ml (**b**) Dox for 24 h, subjected to cell viability assay. Data shown are the mean ± SD. Significant differences were determined by two-tailed unpaired Student’s t-test: ***p < 0.001, NS, not significant. (**c**) U2OS cells were pretreated with or without 200 μM EA for 12 h, and treated with 10 µM NSC87877 (NSC) 1 h before stimulation with 1 µg/ml Dox for the indicated time periods. Cell lysates were subjected to immunoblotting with the indicated antibodies. (**d**) U2OS cells were pretreated with or without 200 µM EA for 12 h and 10 µM NSC87877 (NSC) for the last 1 h, and then stimulated with 0.5 μg/ml Dox for 8 h, followed by incorporation of a ROS-sensitive fluorescent probe DCFH-DA for 30 min. Relative ROS levels are calculated and shown as in Fig. 4d (normalized to the ROS level in cells with no treatment). Significant differences were determined by two-tailed unpaired Student’s t-test: ***p < 0.001; NS, not significant. (**e**) UV sensitivity assay was performed as described in Methods. Nematode worms were fed with *E. coli* expressing dsRNA targeting the indicated genes in the presence or absence of 200 µM EA. Embryonic lethality of the progenies from untreated or UV-irradiated parental worms was measured and represented as mean ± SEM. Sample numbers were as follows from left to right: n = 16, 15, 19, 20, 16, 16, 19, 20, 15, 16, 20, 20, 16, 16, 20, 19, 15, 16, 20, and 19. Significant differences were determined by two-tailed unpaired Student’s t-test: *p < 0.05; NS, not significant.

Tyrosine phosphorylation of mitochondrial proteins, which is mediated by tyrosine kinases residing in mitochondria, including c-Src, has been implicated in the mitochondrial functions and signaling network^28,29^. Previous reports demonstrated that mitochondrial c-Src phosphorylates multiple components of respiratory chain complexes, such as subunit II of COX (cytochrome *c* oxidase)^30^, NDUFV2 (NADH dehydrogenase [ubiquinone] flavoprotein 2) of respiratory complex I, and SDHA (succinate dehydrogenase A) of complex II^31^, and thereby enables efficient energy production for maintaining survival. c-Src-mediated tyrosine phosphorylation is counteracted by tyrosine phosphatases, including Src homology 2 domain-containing protein tyrosine phosphatase 1 (SHP1)^32^, and recent reports have demonstrated that mitochondrial SHP1 negatively regulates mitochondrial electron transport, resulting in mitochondrial ROS production^33,34^. Importantly, it has shown that SHP1 plays a key role in Sab-mediated mitoROS generation^34^. Under normal condition when JNK is inactivated, SHP1 interacts with Sab at the inner side of the mitochondrial outer membrane. Under stress condition when JNK is activated and recruited to Sab, SHP1 is released from Sab, activated, and transferred to the intermembrane face of the inner membrane, where it dephosphorylates its substrates, such as a tyrosine-protein kinase, Src and mitochondrial respiratory complexes, eventually leading to increase in mitoROS generation^34,35^. To examine whether the Sab-SHP1 axis contributes to the EA-mediated pro-apoptotic signaling, we utilized NSC-87877, a potent inhibitor of SHP1 that can efficiently suppress its phosphatase activity^36^. NSC-87877 treatment strongly suppressed EA-mediated enhancement of cell death in several types of cells (Fig. 6a,b), p38/JNK activation (Fig. 6c), and ROS generation (Fig. 6d) induced by Dox treatment, implying that SHP1, a key molecule in Sab-mediated signal transduction, involves in the EA-mediated pro-apoptotic signaling, and that Sab is important for this pro-apoptotic pathway.

### A pro-apoptotic role of EA during DNA damage in an *in vivo* model

To investigate the pro-apoptotic function of EA *in vivo*, we used *Caenorhabditis elegans* (*C. elegans*) nematode worm that is widely used for studying molecular basis underlying DNA damage-induced cell death^37^. Notably, exogenous supplementation of fatty acids to nematode worms is easy, because they can incorporate fatty acids from dietary bacteria seeded on fatty acid-containing agarose growth medium^38^. It is also of note that nematodes are easily applicable for knockdown experiments, since virtually every gene can be systemically knocked down by using dietary bacteria expressing dsRNA that contain the target gene sequence^39^. Taking these advantages, we fed *eri-1* (enhanced RNAi^40^) mutant worms with bacteria expressing either dsRNA targeting genes encoding MOCK (control), REI-1, REI-2 (rab11-interacting protein-1/2, Sab homologs), PTP-2 (protein tyrosine phosphatase-2, SHP1 homolog), or JNK-1 (Jun N-terminal kinase-1, JNK homolog), seeded on agarose growth medium with or without 200 µM EA from L1 (the first larval stage) to Young Adult stage, and subjected them to UV irradiation, which causes germ cell apoptosis resulting in the increase in unhatched eggs laid by the worms^37^. We found that UV-induced embryonic lethality was slightly but significantly increased by EA supplementation, which was suppressed by knocking down either *rei-1*, *rei-2*, *ptp-2*, or *jnk-1* (Fig. 6e), implying an *in vivo* pro-apoptotic role of EA in response to DNA damage through the JNK-Sab-SHP1 axis in *C. elegans*.

## Discussion

In this study, we have shown that TFAs, but not their corresponding *cis* isomers or the saturated fatty acid PA, promote DNA damage-induced apoptosis. Furthermore, among the tested TFAs, promoting effect on DNA damage-induced cell death was observed for EA and LEA, which are most abundant in industrially produced foods (industrial TFAs), but not for TVA, the predominant TFA in ruminant source foods (a ruminant TFA) (Fig. 1a,g). Taken together, these results suggest that industrial TFAs, rather than ruminant TFAs, specifically serve as enhancers for DNA damage-induced apoptosis, which may well explain their specific association with TFA-related disorders, such as CVDs based on epidemiological studies^41,42^. Regarding the actual amount of TFAs in human bodies, the plasma level of EA is ~10 µM and that of LEA is ~2 µM^43^. Although TFA amount in human tissues has not reported yet, in livers of rodents with normal diet, the concentration of EA is estimated to be 1–6 mM^11,44–46^, and that of LEA is estimated to be even higher than EA^46^. Therefore, TFA concentrations utilized in this study (mainly 50–200 µM) are likely to be physiologically relevant.

**Figure 7:**
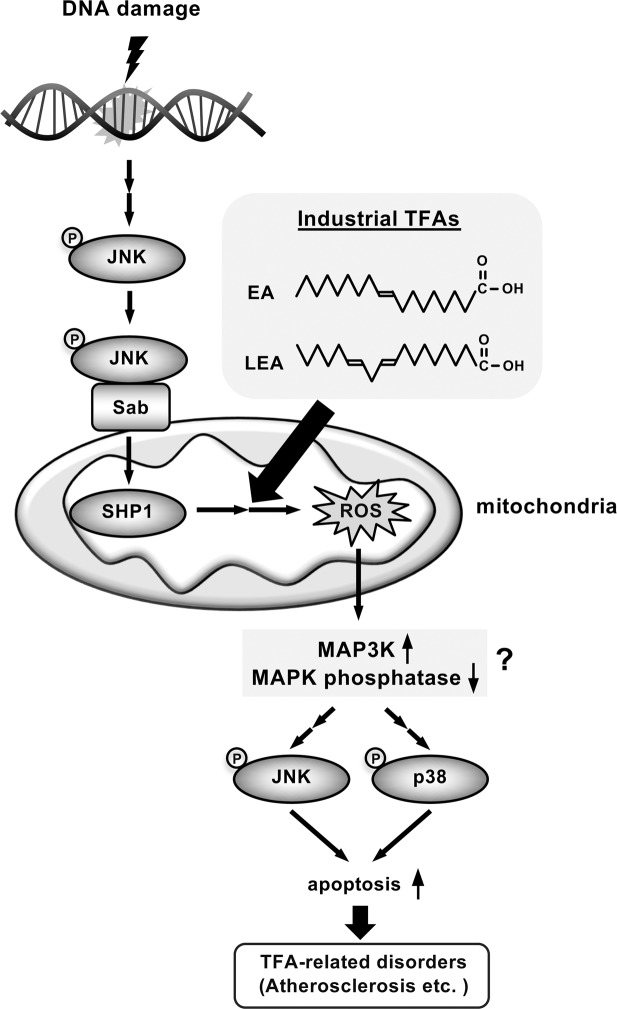
A proposed model for TFA-mediated pro-apoptotic signaling in response to DNA damage. DNA damage induces JNK activation and translocation to the mitochondria via the JNK adaptor Sab that is localized at the mitochondrial outer membrane. After JNK translocation, industrial TFAs, such as EA and LEA, enforce mitochondrial ROS generation through the mitochondrial JNK-Sab signaling involving SHP1. Increased mitochondrial ROS in turn enhance JNK/p38 MAPK activation, possibly by potentiating MAP3K activation or inhibiting MAPK phosphatases, and consequently promote apoptosis, leading to the development and progression of TFA-related disorders, including metabolic syndrome, neurodegenerative disorders, and CVDs.

We have further shown that EA promotes DNA damage-induced cell death by facilitating the mutual enhancement of mitoROS generation and JNK activation mediated by Sab (the mitochondrial JNK-Sab-ROS positive feedback loop) (Figs. 4 and 5). A proposed molecular basis for EA-mediated cytotoxicity based on these results is shown in Fig. 7. Notably, EA did not increase Dox-induced JNK translocation to mitochondria (Fig. 5d), suggesting that the direct target of EA exists downstream of Sab and upstream of ROS generation. EA might affect Sab-dependent regulation of mitochondrial SHP1 localization or activity, which contributes to electron transport inhibition, leading to ROS generation^34,35^. It is also possible that EA targets SHP1 substrates, including mitochondrial Src and respiratory chain complexes, and enforces ROS generation by downregulating their activities^47^. More extensive studies are needed to determine the detailed mechanisms of the EA-mediated pro-apoptotic signaling in mitochondria. Increased DNA damage-induced mitoROS by EA causes hyperactivation of p38 and JNK MAP kinases independently of ASK1, although ASK1 is well-known as a ROS-activated MAP3 kinase^48–51^ and involves EA-mediated promotion of extracellular ATP-induced apoptosis^11^. Another ROS-activated MAP3 kinase, such as MAP/ERK kinase kinase 1 (MEKK1)^52^ or mixed lineage kinase 3 (MLK3)^53^, may be involved in EA-mediated hyperactivation of MAP kinases; otherwise, ROS-induced inactivation of MAP kinase phosphatase, such as dual specificity phosphatase 1 (DUSP1)^54^ or protein phosphatase 2 A (PP2A)^55,56^, can possibly contribute to it.

To date, the pivotal roles of JNK in DDRs have been established^57^. In response to DNA damage, JNK is activated by diverse mechanisms, including activation of upstream molecules, such as RAS-related C3 botulinus toxin substrate 1 (Rac1) and TGF-β-activated kinase 1 (TAK1), and downregulation of DUSP1, and plays a key role in inducing apoptosis signaling by DNA injury^57,58^. Since DNA damage-induced cell death is associated with a variety of disorders, such as atherosclerosis^59,60^ and neurodegenerative diseases^61^, TFAs may exacerbate these disorders by enhancing JNK activation and subsequent apoptosis via the mitochondrial JNK-Sab-ROS positive feedback loop. Supporting this idea, mouse studies have revealed that JNK1 is required for epithelial cell apoptosis at atherosclerotic site^62^, and that, intriguingly, TFA-containing diet significantly augments laser irradiation-induced thrombus formation in the carotid artery, accompanied with increase in JNK phosphorylation^63^. Meanwhile, importantly, JNK also regulates other DDRs, such as cell cycle arrest and inflammation^57^, which raises the possibility that TFAs may promote slightly multiple DDRs thorough the mitochondrial JNK-Sab-ROS positive feedback loop, and that TFA-mediated promotion of multiple DDRs may ultimately provoke the development and progression of TFA-related disorders. Probably, TFAs dysregulate the balance between DDRs-promoted cell survival and apoptotic cell death. These assumptions should be addressed in future studies.

The mitochondrial JNK-Sab-ROS activation loop has been implicated in various types of cytotoxicity, besides DNA damage-induced cell death^26,64^: anisomycin (a widely used JNK activator)-induced cell death^24^; hepatotoxicity by acetaminophen, TNF-α^65^, ER stress^66^, and palmitic acid^27^; neurotoxicity by 6-hydroxydopamine^67^; cardiotoxicity by ischemia/reperfusion^68^ and imatinib mesylate^69^. It should also be noted that, although the contribution of Sab to JNK activation has been shown in limited types of cytotoxicity and disease models, numerous studies have demonstrated the pleiotropic functions and pivotal roles of JNK in the pathogenesis of diseases linked with TFAs, such as inflammatory diseases, metabolic syndrome, neurodegenerative disorders, and CVDs^70–72^. We have shown that Sab-knockdown suppressed DNA damage-induced cell death (Fig. 5b) and ROS generation (Fig. 5c) in the presence of EA, but apparently not those in the absence of EA, suggesting that TFAs have a potential to induce activation of the mitochondrial JNK-Sab-ROS loop, which is not activated under normal stress condition without TFAs. Therefore, TFAs may also promote or induce the mitochondrial JNK-Sab-ROS activation loop in stress conditions other than DNA damage, drive cell death and inflammation, and thereby result in TFA-related disorders. Supporting this notion, we confirmed that EA, but not OA, substantially promotes cell death induced by anisomycin that is a protein synthesis inhibitor, commonly used as a stimulus for activating the mitochondrial JNK-Sab-ROS axis^24^ (see Supplementary Fig. S3). Notably, a cell membrane-permeable peptide trans-activator of transcription (tat)-Sab_KIM1_, mimicking JNK binding motif in Sab, has been developed for specifically interrupting the interaction of JNK and Sab without affecting kinase activity of JNK^73^. Therapeutic effectiveness of tag-Sab_KIM1_ has been demonstrated in various *in vitro* and *in vivo* disease models associated with the mitochondrial JNK-Sab-ROS axis^74^, implicating its potential use for treatment of TFA-related diseases including CVDs. Thus, our study provides a crucial molecular basis for understanding and uncovering the common pathogenetic mechanisms of and novel therapeutic targets for diverse TFA-related disorders, including atherosclerosis, cardiovascular diseases, and neurodegeneration.

## Methods

### Reagents

Doxorubicin (Dox) was purchased from Sigma. Cisplatin, anisomycin, propyl gallate (PG), SP600125, N-acetylcysteine (NAC) and U0126 were purchased from Wako. SB203580, Etoposide, Apocynin (Apo) and mitoTEMPO (MT) were purchased from Santa Cruz. NSC87877 and wortmannin were purchased from Cayman.

### Cell culture

U2OS, HeLa and HEK293T cells, and RAW264.7 cells were cultured in Dulbecco’s Modified Eagle’s medium and RPMI 1640 medium, respectively, containing 10% heat-inactivated fetal bovine serum and 1% penicillin-streptomycin solution in 5% CO2 at 37 °C. HUVECs (kindly provided by Dr. Junken Aoki, Tohoku University) were cultured in HuMedia-EG2 with a growth additive set.

### siRNA knockdown

siRNA targeting human *Sab* was obtained from GeneDesign. U2OS cells were transfected with 10 nM non-targeting siRNA pool (Dharmacon) as control or *Sab* siRNA using Lipofectamine RNAiMAX Transfection Reagent (Invitrogen), according to the manufacturer’s instructions. *Sab* siRNA sequence was 5′-GGAGCGAGCUGGUGCAUAA-3′.

### Preparation and treatment of fatty acids

Fatty acids including PA, OA (Nacalai tesque), EA (Sigma), LA, LEA (Cayman Chemical), *cis* CVA and TVA (Olbracht Serdary Research Laboratories) were prepared as described previously^75^. Briefly, fatty acids were dissolved in 0.1 N NaOH at 70 °C, and then conjugated with fatty acid-free BSA (Wako, pH 7.4) at 55 °C for 10 min to make 5 mM BSA-conjugated fatty acid stock solutions containing 10% BSA. Cells were treated with various concentrations of BSA-conjugated fatty acids by diluting stock solutions in medium without fetal bovine serum (final BSA concentration was set to 1%).

### Immunoblot analysis

Cells were lysed in ice-cold lysis buffer containing 20 mM Tris-HCl, pH 7.4, 150 mM NaCl, 1% Triton-X100, 10% Glycerol, and 1% protease inhibitor cocktail (Nacalai tesque). Alternatively, in Fig. 2a,e, cells were lysed in another ice-cold lysis buffer containing 22 mM Tris-HCL, pH 7.4, 150 mM NaCl, 6 mM EDTA-2Na pH 8.0, 0.6% Triton-X100, 1% Sodium deoxycholate, 0.4% NP-40, 0.04% SDS, and 1% protease inhibitor cocktail, for extracting nuclear proteins. After centrifugation, the cell extracts were resolved by SDS-PAGE, and were analyzed as described previously^14^. In Fig. 2c, nuclear extraction was performed as described previously^76^, whereas in Figs. 2d and 5d, mitochondria were isolated as described previously^24^, and subjected to immunoblot analysis. The antibodies used for immunoblotting were against phospho-p38, p38, phospho-JNK, JNK, caspase-3, caspase-9, p53 (Cell Signaling), β-actin, GAPDH (Wako), γH2AX, H2AX, Lamin A/C (Santa cruz), Sab, and Cox4 (Proteintech). The blots were developed with ECL (Merck Millipore), and detected with ChemiDoc Touch Imaging System (BioRad). All experiments were performed at three independent times. The relative band intensity was calculated using Image Lab software (BioRad), normalized to that of the corresponding loading control, and shown as mean ± SD. Significant differences were determined by one-way ANOVA, followed by Tukey-Kramer test: *p < 0.05; **p < 0.01; ***p < 0.001; NS, not significant (See Supplementary Fig. 5).

### Cell viability assay

RAW264.7, U2OS, Hela cells and HUVECs were seeded on 96-well plates. After any stimulation or treatment, cell viability was determined using Cell Titer 96 Cell Proliferation Assay (Promega), according to the manufacturer’s protocol. The absorbance was read at 492 nm using a microplate reader (Multiskan Ascent, Thermo). Data are normalized to control without stimulus, unless noted otherwise.

### DNA fragmentation assay

DNA fragmentation assay was performed as described previously^14^. Briefly, stimulated cells were collected and suspended with lysis buffer (20 mM Tris-HCl, pH7.5, 10 mM EDTA, and 0.5% Triton X-100), and the cell lysates were incubated at room temperature for 10 min, followed by centrifugation at 12,000 g for 10 min. The supernatants were incubated with 0.2 mg/ml proteinase K and 0.1 mg/ml RNase A for 1 h at 42 °C, purified with phenol/chloroform extraction and ethanol precipitation, and separated on an agarose gel.

### Bioimaging and quantification of ROS

RAW264.7 and U2OS cells were seeded on glass plates. After stimulation, cells were treated with 10 μM DCFH-DA (Sigma) or 5 µM MitoSOX Red (Invitrogen) for 30 min at 37 °C. Intracellular ROS generation was observed using a Zeiss LSM800 laser confocal microscope (Carl Zeiss) and the images were processed with Zen software. Data are shown as mean ± SD of relative fluorescence intensity from three different fields of view, which was calculated by dividing total fluorescence (background was subtracted) by cell numbers using Image J.

### Immunocytochemistry

Immunocytochemistry was performed as described previously^51^, using antibodies against γH2AX (Santa cruz), 8-OHdG (Nikken Seil), and β-actin (Proteintech). The immunostained samples were observed with either Zeiss LSM800 or Olympus Fluoview FV1000 confocal fluorescence microscope. 8-OHdG intensity was measured in nuclear (DAPI positive areas) and non-nuclear (β-actin positive areas outside of DAPI positive areas) for 20 cells per sample using Image J, and data are shown as mean ± SEM.

### Generation of knockout cell lines

ASK1*-knockout RAW264.7 cells were generated previously*^11^. *p53- and TNFR1*-knockout cells were generated using the CRISPR/Cas9 system^77,78^. guide RNAs (gRNAs) were designed to target exon 5 of *p53* gene (5′-ACCATGAGCGCTGCTCAGAT-3′) and exon 3 of *TNFR1* gene (5′-GGGGCAGGATACGGACTGCA-3′) using CRISPRdirect^79^. gRNA-encoding oligonucleotides were cloned into lentiCRISPRv2 plasmid^80^, and the plasmids were transfected with HEK293T cells together with a packaging plasmid psPAX2 and an envelope plasmid pVSV-G. The virus-containing supernatants were collected and used for infecting RAW264.7 and U2OS. DNA sequences around the gRNA target site of *TNFR1* gene in WT and two KO U2OS cell lines are as follows (deletion: dash, insertion: under bar); WT: 5′-ggggcaggatacggactgcagggagtg-3′, KO#1: 5′-ggggcaggatacggacttgcagggagtg-3′ and 5′-ggggcaggatacgga-tgcagggagtg-3′, KO#2: 5′-ggggcaggatacggacttgcagggagtg-3′.

### Culture methods and strains of *C. elegans*

Maintenance of *C. elegans* was carried out as described previously using normal growth medium^81^. An enhanced RNAi strain *eri-1(mg366)*^40^ mutant was backcrossed with wild-type strain Bristol N2 at five times before analysis. *Escherichia coli* (*E. coli*) OP50 was used as the food source. Feeding RNAi was performed as described previously^82^, using the HT115 bacterial strain transformed with either L4440 empty RNAi vector (MOCK), RNAi vector targeting *rei-1, rei-2* (from Ahringer library^83^)*, ptp-2*, or *jnk-1* (from ORFeome-RNAi library^84^). Target sequences in RNAi vectors were verified by sequence analyses.

### UV sensitivity assay

To synchronize the growth of *eri-1* mutant worms, adult worms were treated with 5% NaClO (Nakalai) solution. Synchronized L1 worms were transferred onto feeding RNAi plates with agarose medium containing 0 or 200 µM EA. After 2 days of culture (at Young Adult stage), worms were either left untreated or irradiated with 20 mJ/cm^2^ UV-C using CL-1000 Ultraviolet Crosslinker (UVP), and transferred one by one onto new RNAi plates. One day later, parental worms (P0) were eliminated and after one more day, hatched F1 larvae and unhatched eggs were counted. Embryonic lethality = unhatched eggs/(unhatched eggs + F1 larvae (hatched eggs)).

## Supplementary information

Supplementary Figures.
