# Supplementary material for: trans-Fatty acids facilitate DNA damage-induced apoptosis through the mitochondrial JNK-Sab-ROS positive feedback loop
Source: Sci Rep. 2020 Feb 17;10:2743. doi: 10.1038/s41598-020-59636-6 (PMC7026443; doi:10.1038/s41598-020-59636-6)
Supplement: Supplementary file 1 — Supplementary Figures. [file 41598_2020_59636_MOESM1_ESM.pdf]

Supplementary Information

trans-Fatty acids facilitate DNA damage-induced apoptosis through the mitochondrial JNK-Sab-ROS positive feedback loop

Yusuke Hirata, Aya Inoue, Saki Suzuki, Miki Takahashi, Ryosuke Matsui, Nozomu Kono, Takuya Noguchi, and Atsushi Matsuzawa

Included materials

・**Figure S1.**

・**Figure S2.**

・**Figure S3.**

・**Figure S4.**

・**Figure S5.**

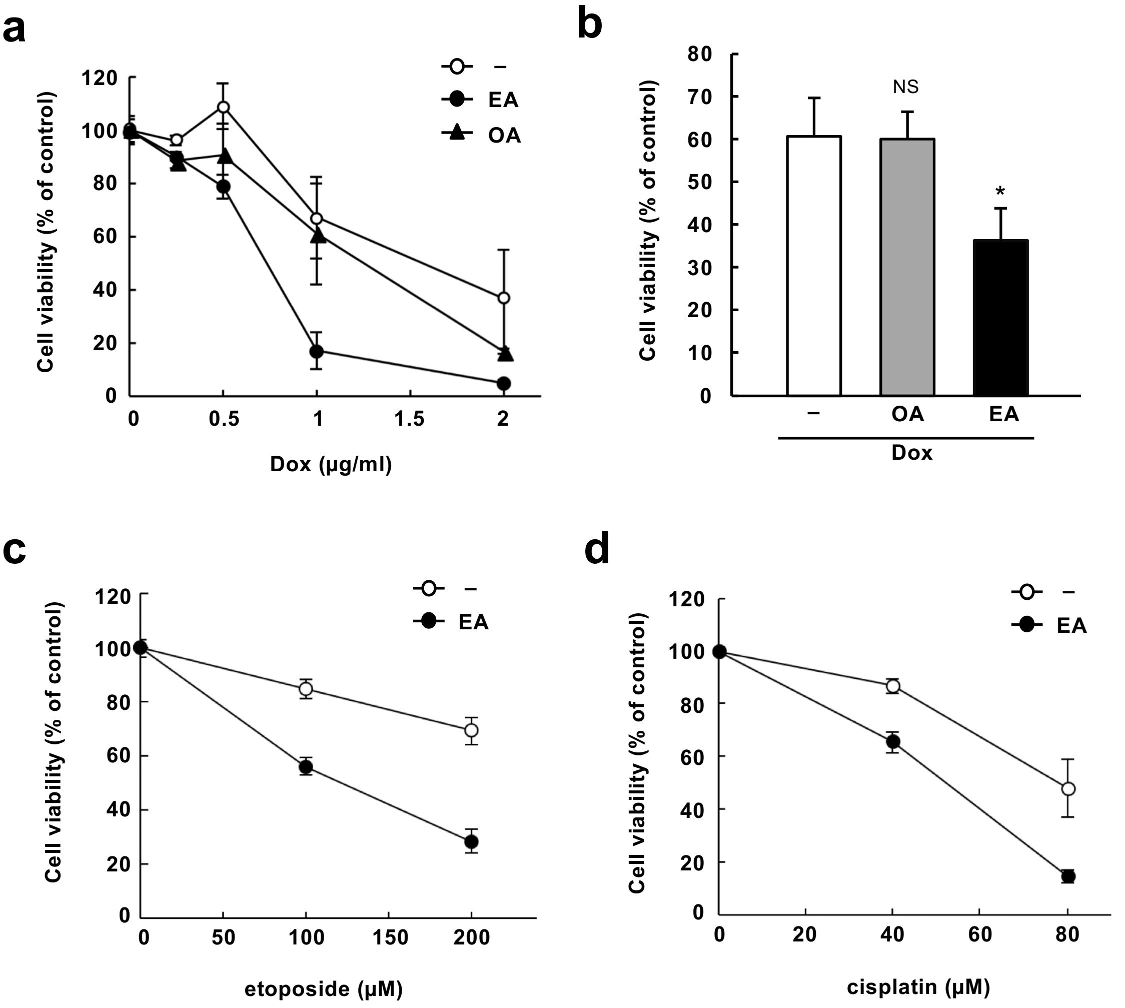

Figure S1. EA promotes DNA damage-induced cell death in various experimental conditions
(a and b) HeLa cells (a) and HUVECs (b) were pretreated with or without 200 μM OA or EA for 12 h, and then stimulated with various concentrations of Dox (a) or 0.5 μg/ml Dox (b) for 24 h (a) or 36 h (b), subjected to cell viability assay. (c and d) RAW cells were pretreated with or without 200 μM EA for 12 h, and then stimulated with various concentrations of etoposide (c) or cisplatin (d) for 24 h, subjected to cell viability assay. Data shown are the mean ± SD. In Fig. S1b, significant differences were determined by one-way ANOVA, followed by Tukey-Kramer test; *p < 0.05; NS, not significant (versus control cells without fatty acid)

Hirata et al., Fig. S1.

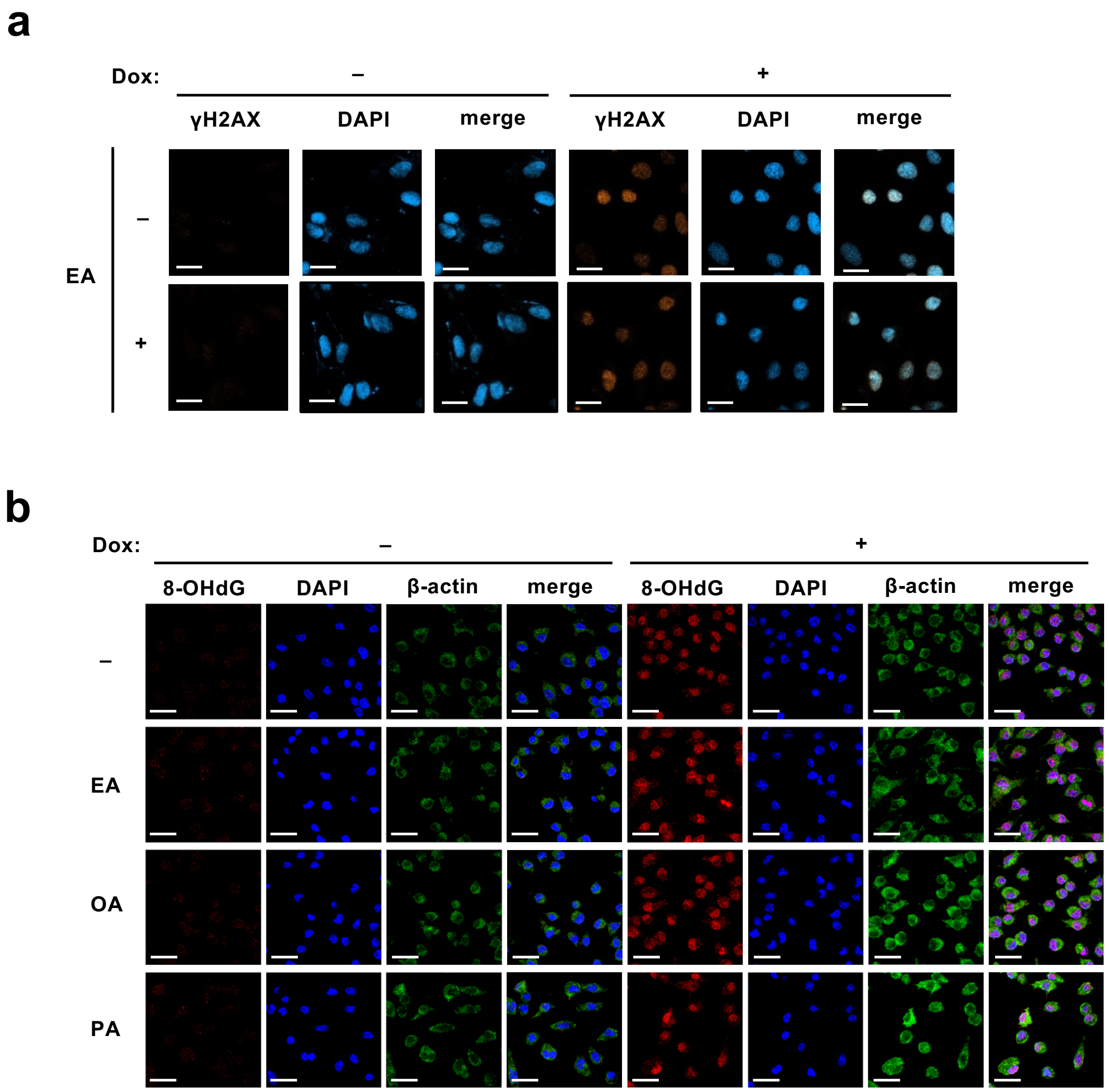

Figure S2. Immunocytochemistry for DNA damage markers
(a and b) U2OS cells (a) and RAW264.7 cells (b) were pretreated with the indicated fatty acids, and treated with Dox for 0 or 2 hours, subjected to immunocytochemistry using antibodies against γH2AX (red) (a), 8-OHdG (red) and β-actin (green) (b). Scale bar, 25 µm.

Hirata et al., Fig. S2.

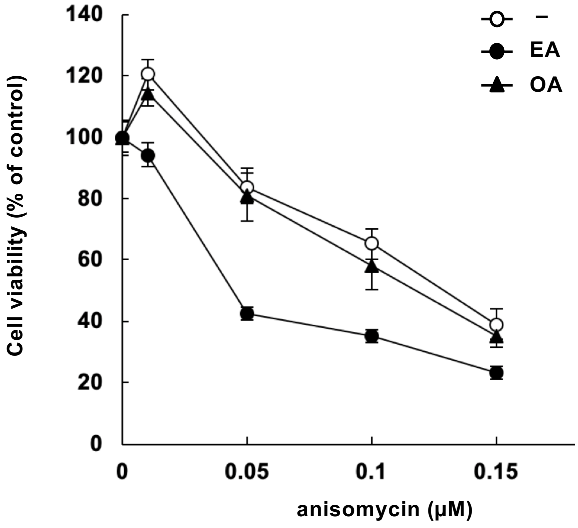

Figure S3. EA specifically promotes anisomycin-induced cell death
RAW cells were pretreated with or without 200 μM OA or EA for 12 h, and then stimulated with various concentrations of anisomycin for 24 h, subjected to cell viability assay.

Hirata et al., Fig. S3.

a

Fig. 1c

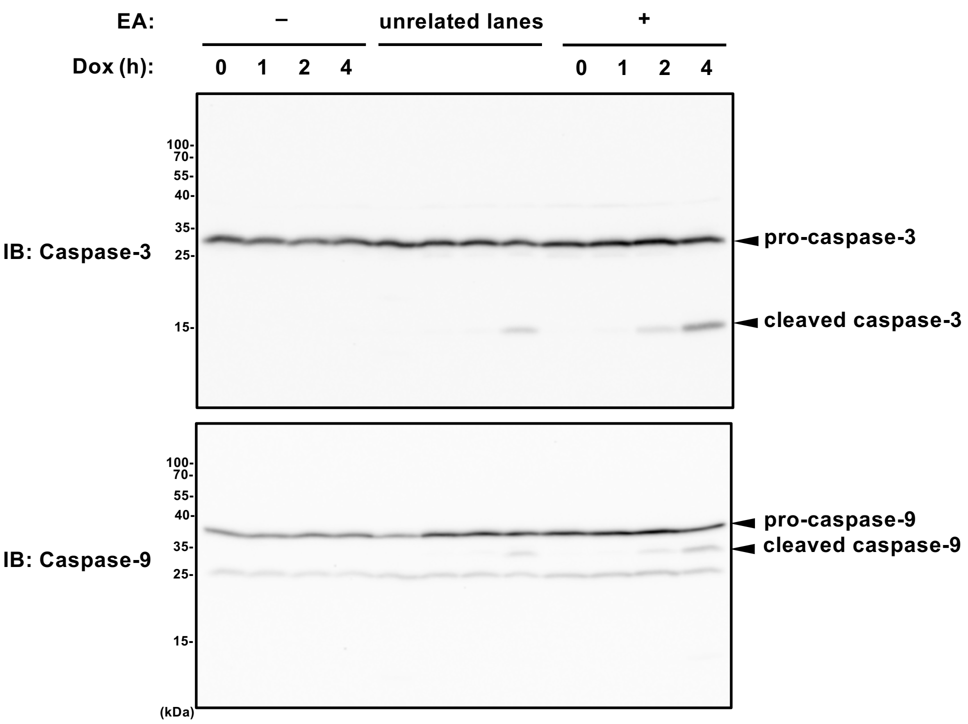

b

Fig. 2a

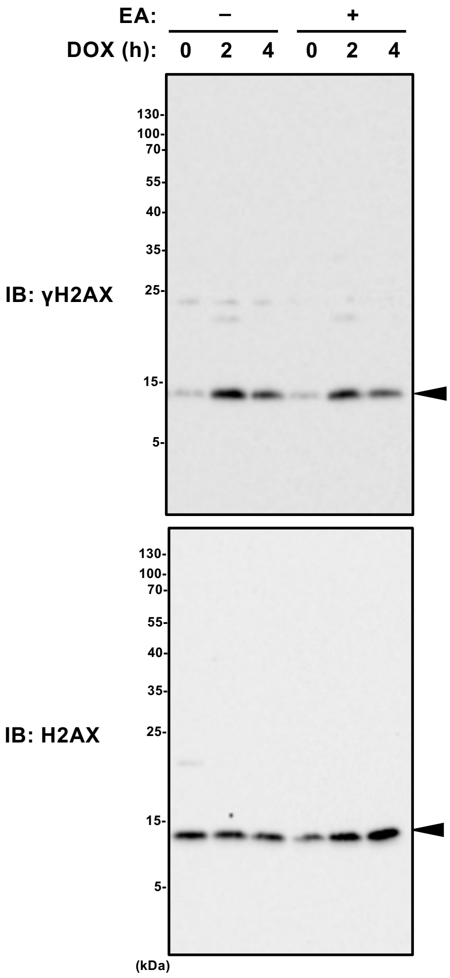

Hirata et al., Fig. S4.

c

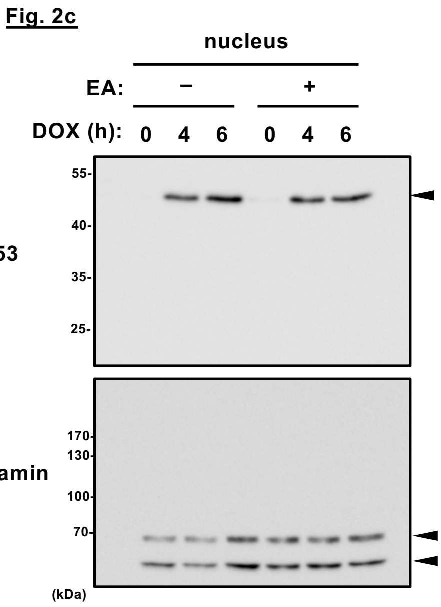

d

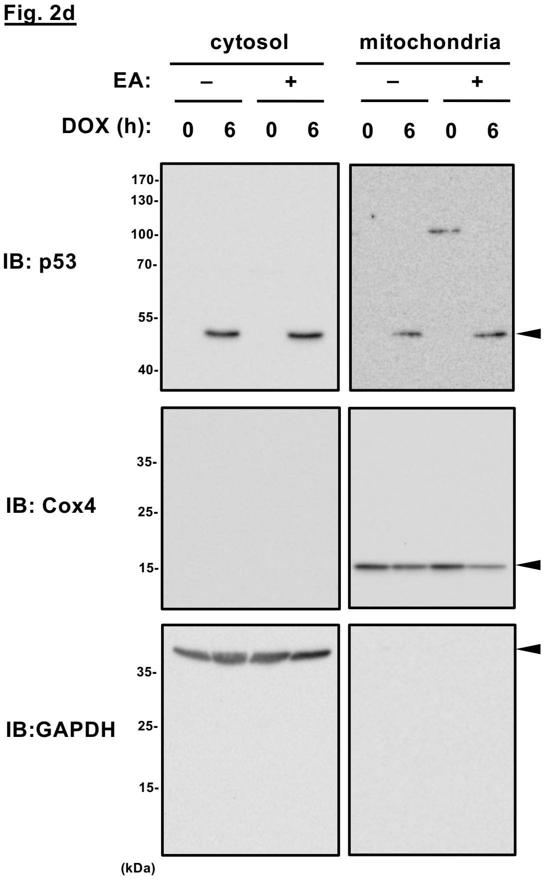

e

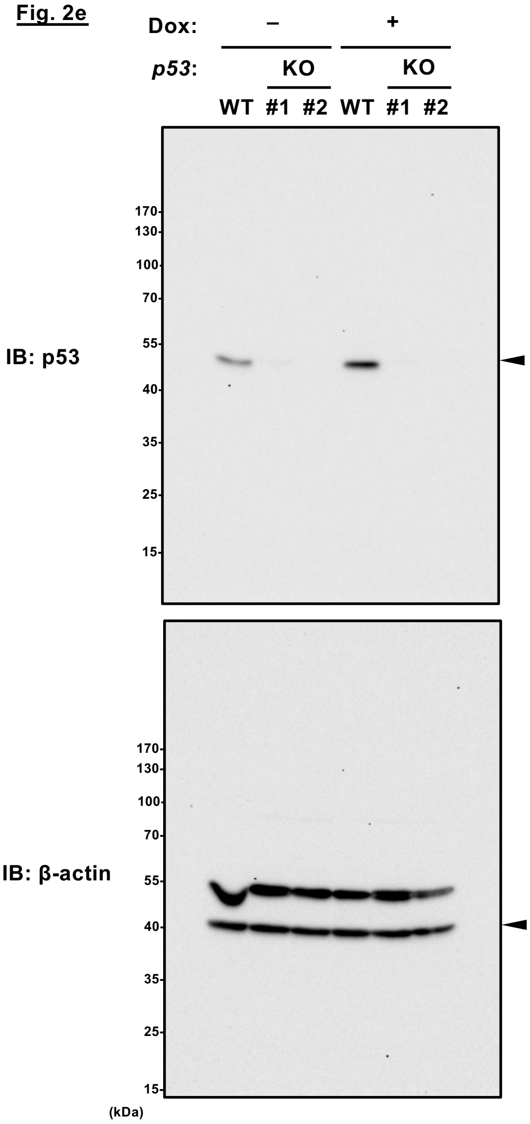

Hirata et al., Fig. S4.

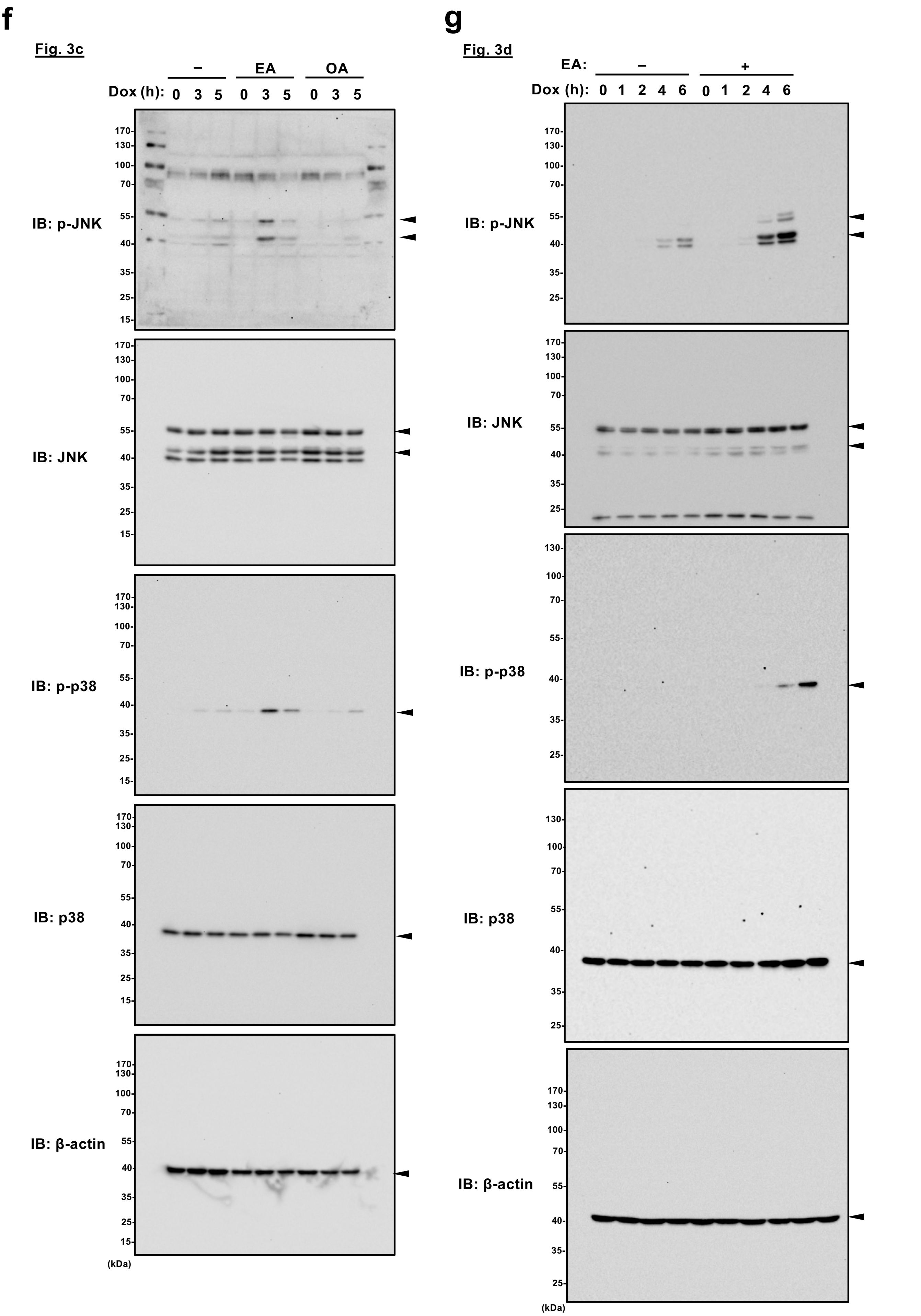

Hirata et al., Fig. S4.

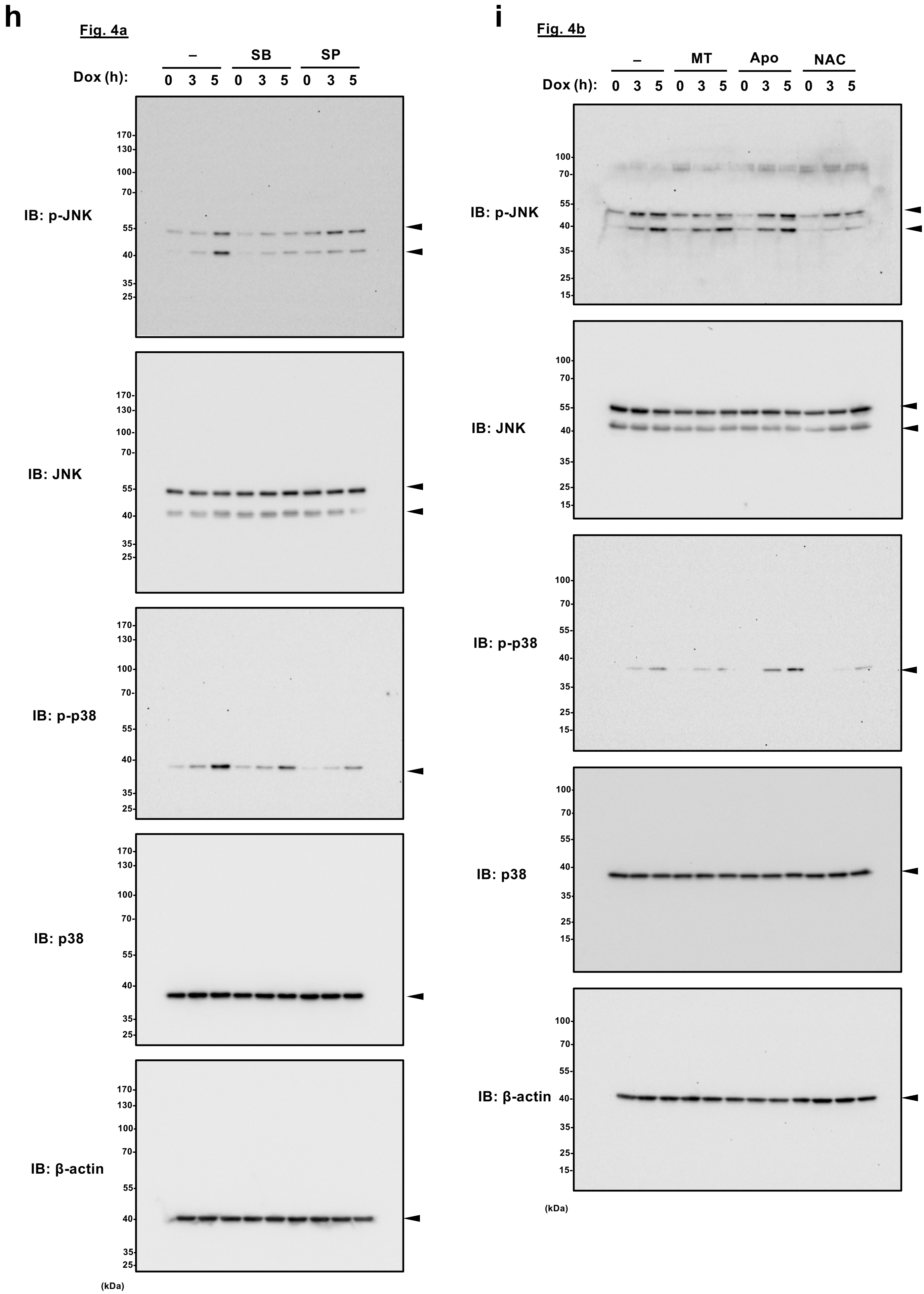

Hirata et al., Fig. S4.

j

Fig. 5a

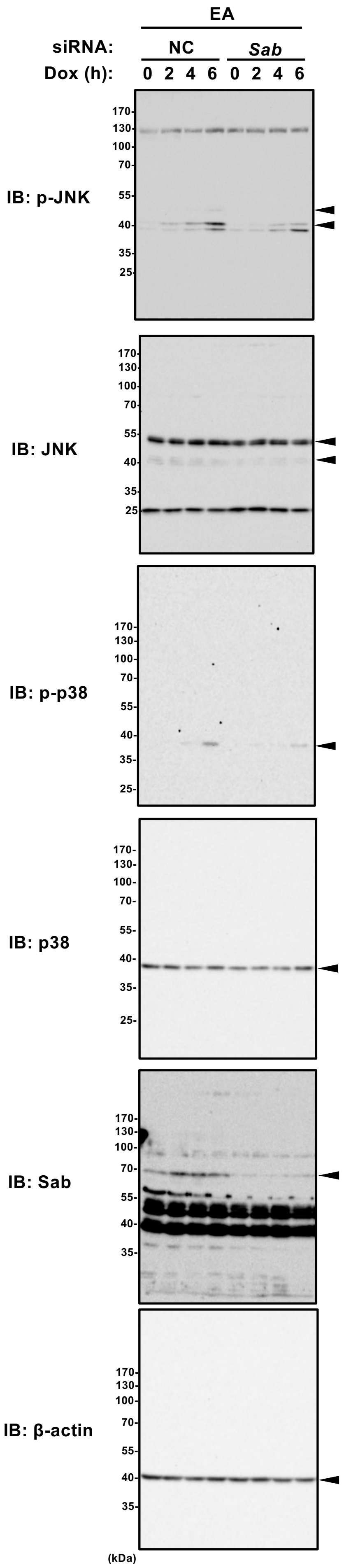

k

Fig. 5d

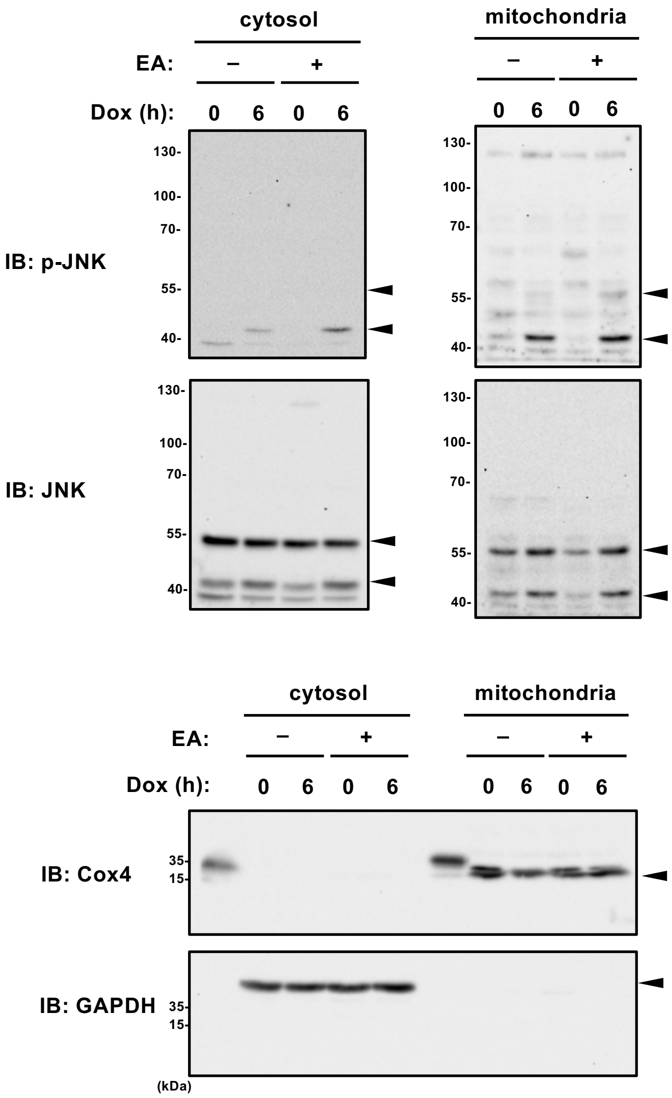

Hirata et al., Fig. S4.

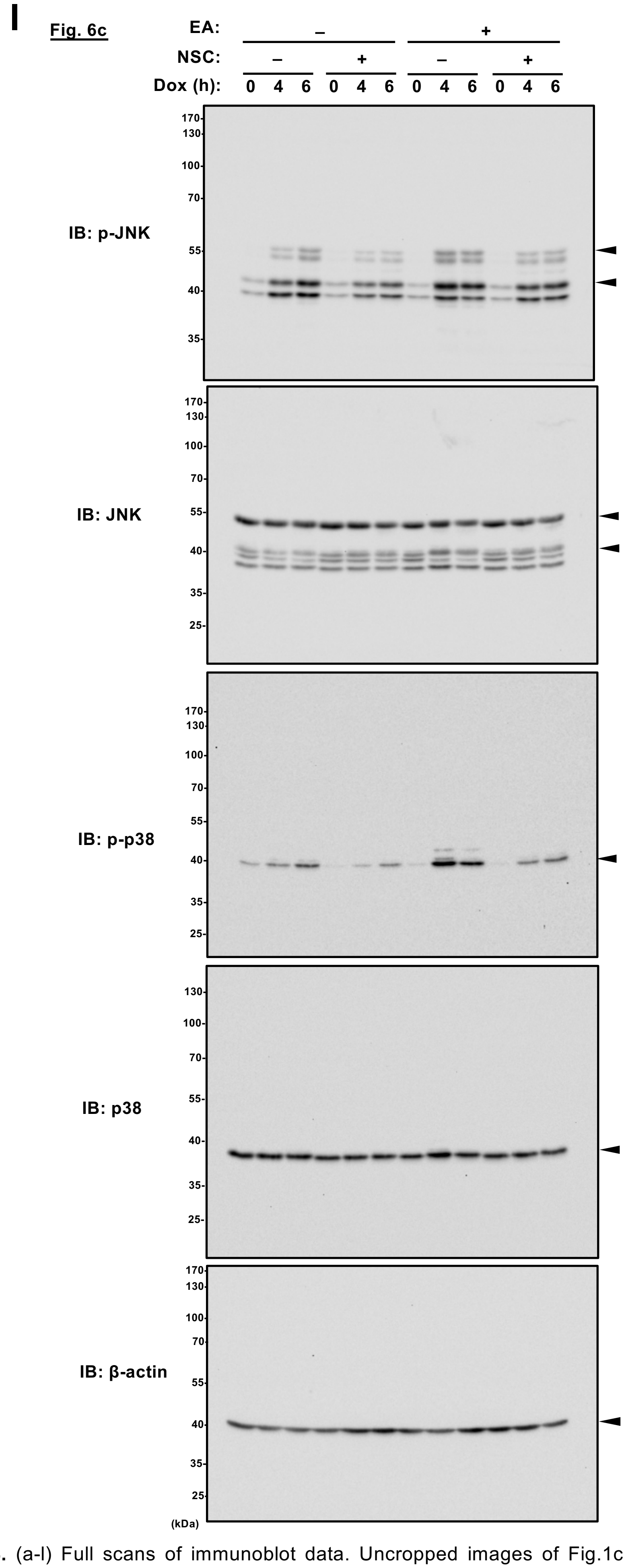

Figure S4. (a-l) Full scans of immunoblot data. Uncropped images of Fig.1c (a), Fig.2a (b), Fig.2c (c), Fig.2d (d), Fig.2e (e), Fig.3c (f), Fig.3d (g), Fig.4a (h), Fig.4b (i), Fig. 5a (j), Fig 5d (k) and Fig 6c (l).

Hirata et al., Fig. S4.

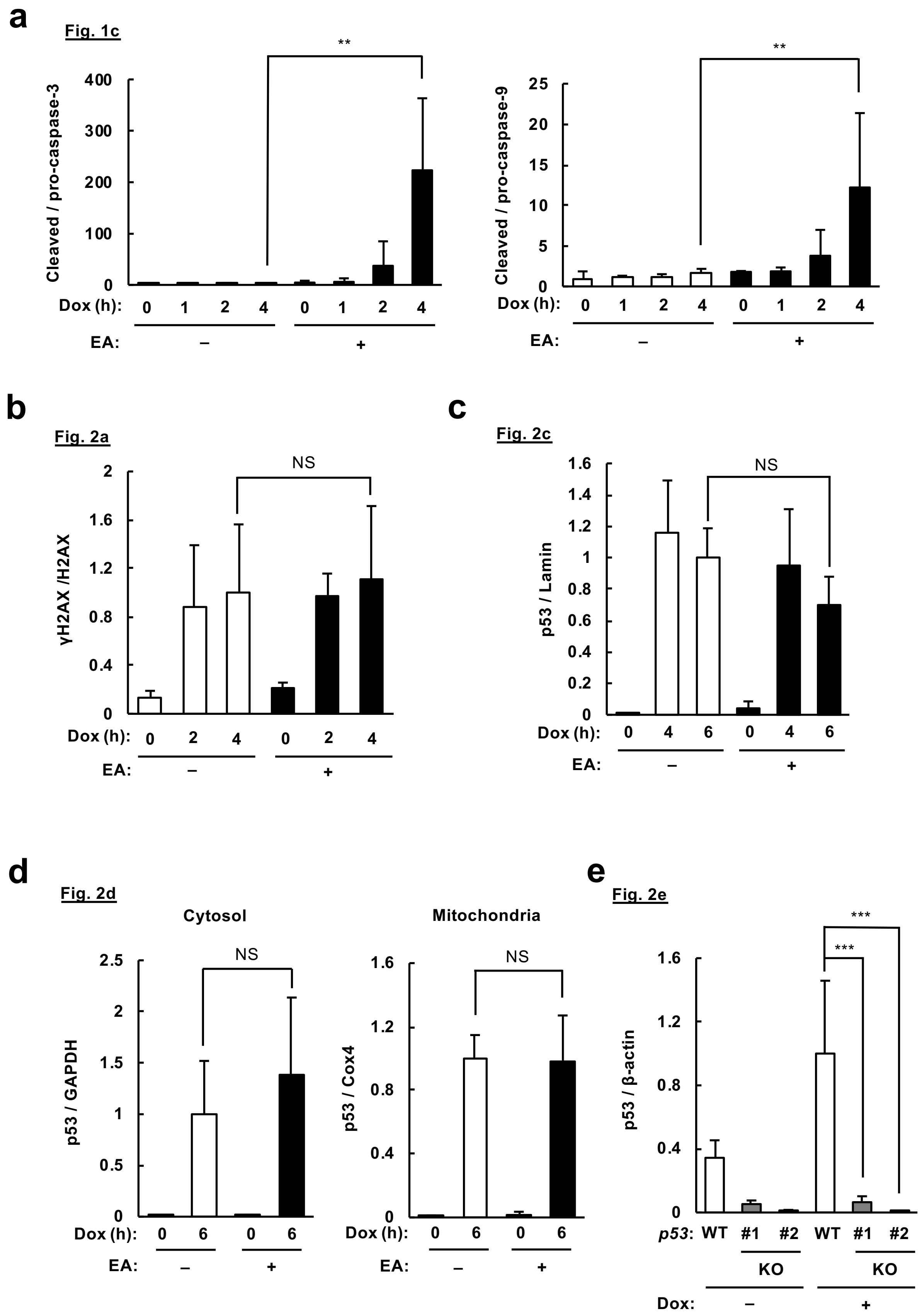

Hirata et al., Fig. S5.

f Fig. 3c

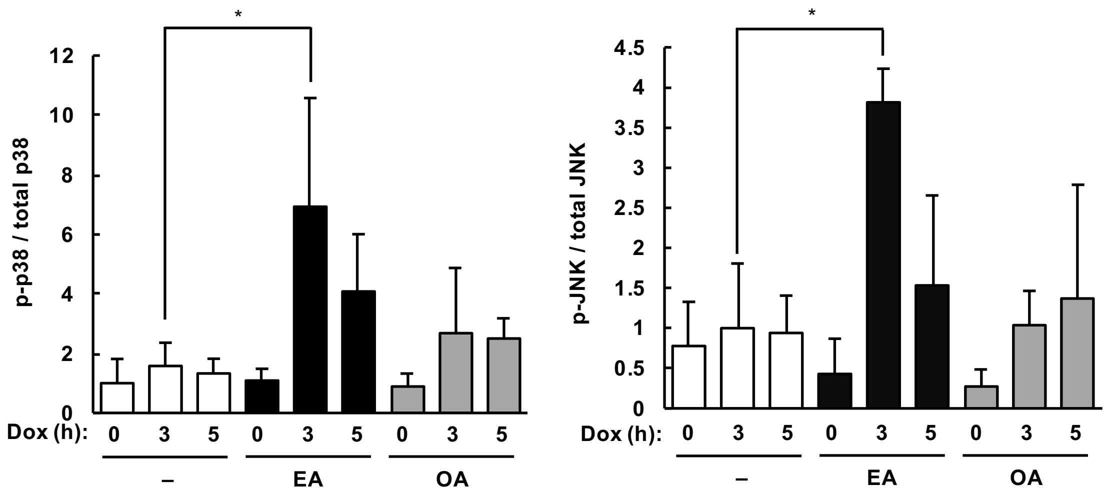

g Fig. 3d

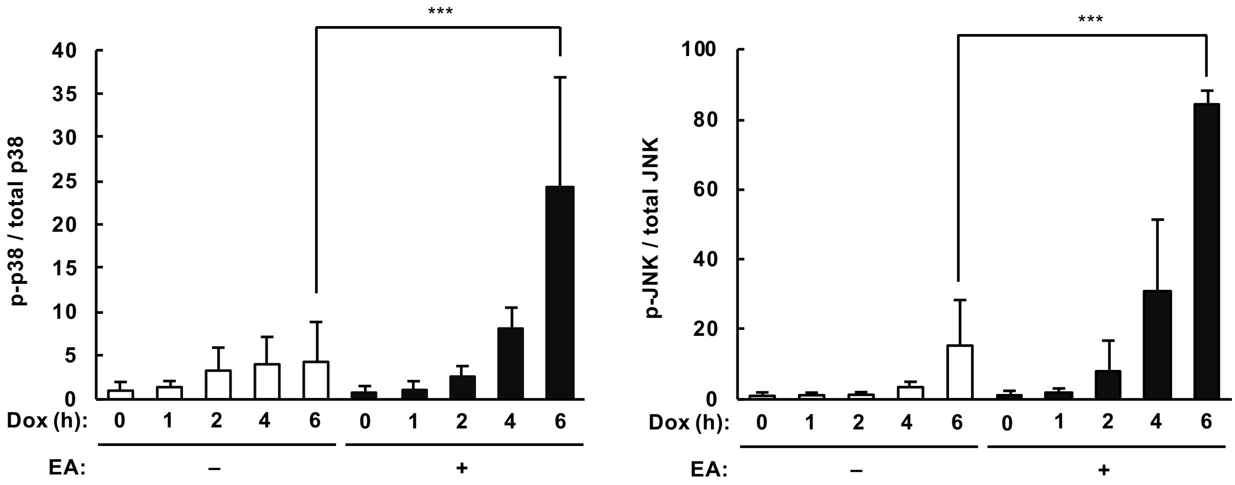

h

Fig. 4a

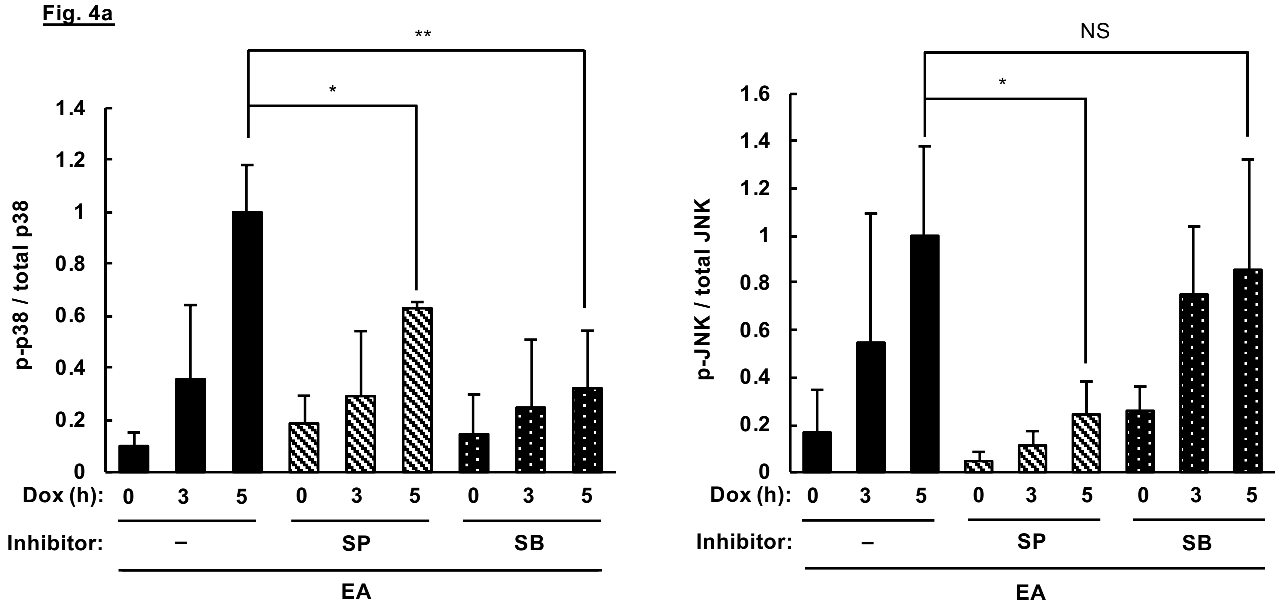

Hirata et al., Fig. S5.

i

Fig. 4b

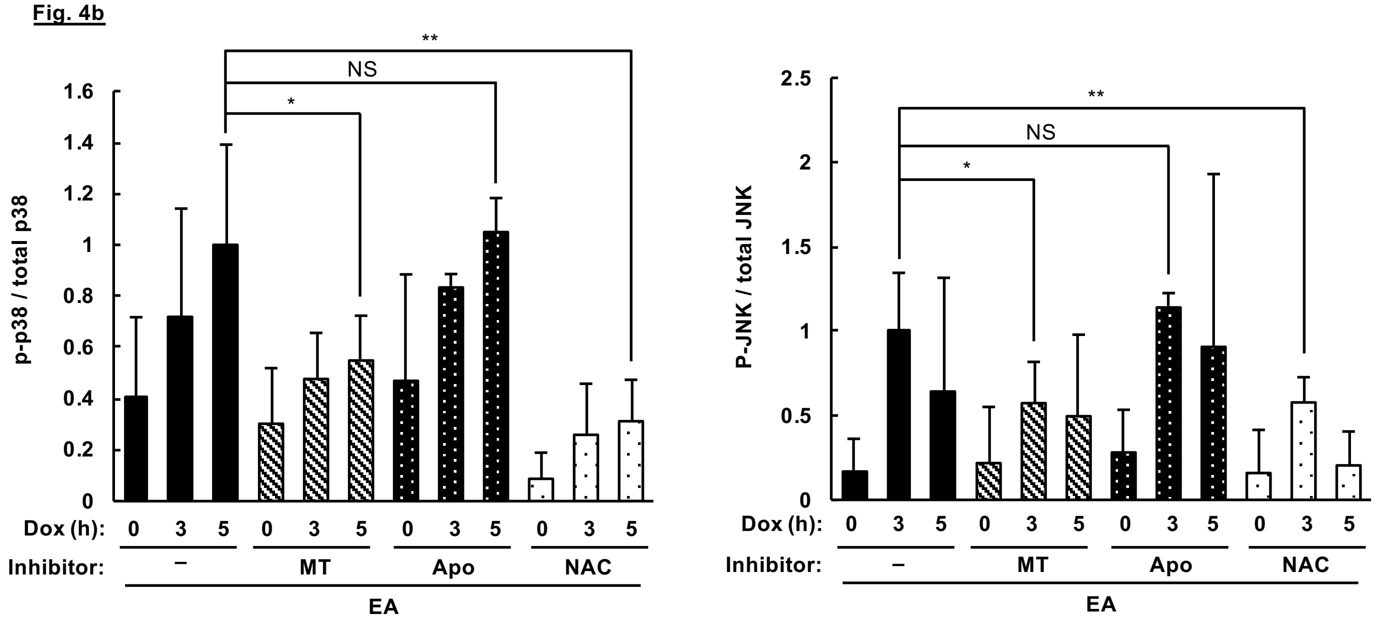

j

Fig. 5a

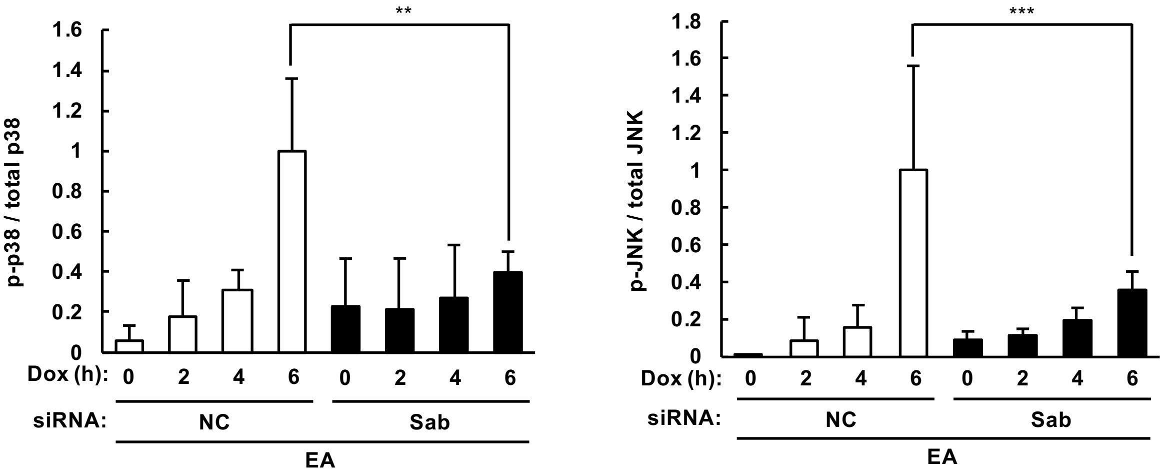

k

Fig. 5d

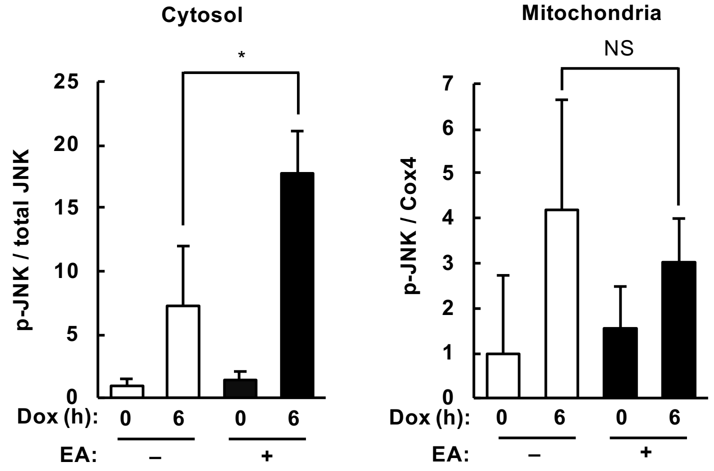

Hirata et al., Fig. S5.

l

Fig. 6c

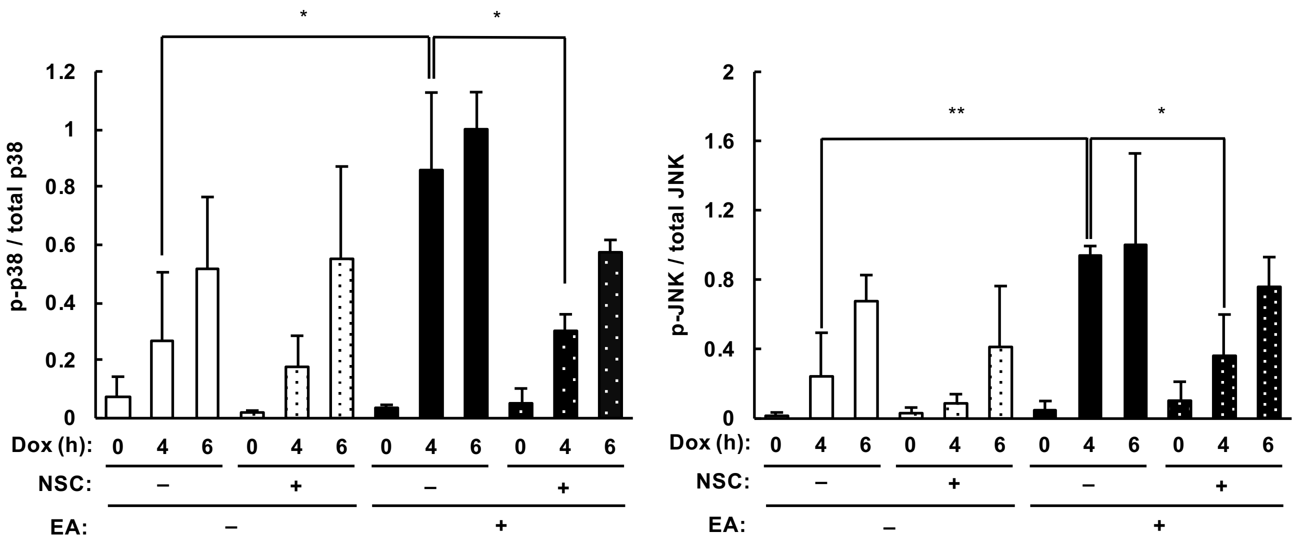

Figure S5. (a-k) Quantified relative band intensity of immunoblot data are shown as mean ± SD (n = 3): Fig. 1c (a), Fig. 2a (b), Fig. 2c (c), Fig. 2d (d), Fig. 2e (e), Fig.3c (f), Fig. 3d (g), Fig. 4a (h), Fig. 4b (i), Fig. 5a (j), Fig. 5d (k) and Fig. 6c (l).

Hirata et al., Fig. S5.
